# GC–MS analysis, molecular docking, and pharmacokinetic studies of *Multidentia crassa* extracts’ compounds for analgesic and anti-inflammatory activities in dentistry

**DOI:** 10.1038/s41598-023-47737-x

**Published:** 2024-01-22

**Authors:** Ibrahim Chikowe, King David Bwaila, Samuel Chima Ugbaja, Amr S. Abouzied

**Affiliations:** 1 Pharmacy Department, Kamuzu University of Health Sciences (KUHES), Blantyre, Malawi; 2 Pharmacy Department, Malawi College of Health Sciences, Zomba, Malawi; 3 The Department of Population Science, African Health Research Institute (AHRI), Durban, South Africa; 4 Department of Pharmaceutical Chemistry, College of Pharmacy, University of Hail, 81442 Hail, Saudi Arabia; 5 Department of Pharmaceutical Chemistry, National Organization for Drug Control and Research (NODCAR), Giza, 12553 Egypt

**Keywords:** Computational biology and bioinformatics, Drug discovery, Chemistry

## Abstract

Plant extracts have been useful for oral health or dentistry. However, only a few evidence-based justifications exist. This study evaluated *Multidentia crassa* (Hiern) Bridson & Verdc, one of the oral health-used plants in Malawi. Gas chromatography-mass spectrometry (GC-MS) and Fourier transform infrared (FT-IR) identified the extracts’ compounds. The pharmacokinetics of the identified compounds were studied using pkCSM and SwissADME, and molecular docking studies were used to identify potential drug candidates for oral health by predicting the binding affinity of the compounds to cyclooxygenases, interleukin-1 beta receptors, odontoblast cold sensor proteins, and purinergic receptor P2X3. FT-IR analysis showed characteristic peaks of phenols, carboxylic acids, alkenes, alkyl halides, amines, esters, ethers, aromatics, and lipids. GC–MS results showed the presence of 58 bioactive phytocompounds, some of which have various pharmacological activities relevant to oral health. Molecular docking further validated stigmastan-3,5-diene’s potency for analgesic and anti-inflammatory purposes. Based on a literature review, this is the first report on the bioactive compounds of *M. crassa* extracts showing analgesic and anti-inflammatory effects. This study's results can lead to new herbal and conventional medicines. Therefore, we recommend in vivo and in vitro studies to elucidate the pharmacological effects of the plant extracts.

## Introduction

Approximately 3.5 billion people worldwide are affected by dental or oral health diseases. The commonly reported oral health problems include toothache, gum inflammation, canker sores^1^, bleeding, infections (bacteria, fungi, viruses), cancer^2–5^, cold sores, caries, tooth abscess, oral ulcers, and plaque accumulation^6,7^. However, many oral health problems are characterized by toothache or orofacial pain that originates from the dental element and/or adjacent structures. The World Health Organisation (WHO) has identified toothache as one of the priority issues in oral health because it is a common marker for almost all oral health problems^8^. A dental health survey carried out in Malawi by Msyamboza et al. in 2016 showed that 37.4% of the respondents had dental caries, 35.2% had missing teeth, 6.5% had filled teeth, 23.5% had bleeding gums, and 35.2% brushed teeth twice a day^9^. The WHO projected an increase in dental or oral health problems, especially in low and middle-income countries (LMICs), due to low access to oral health services, inadequate exposure to fluoride in water and oral hygiene products, high-risk factors (tobacco and alcohol), and unhealthy diets high in free sugars, which are exacerbated by growing urbanization and changes in living conditions^10^. Despite the overwhelming number of people affected by oral health problems, there are only a few treatment options available, and those available options are mostly not part of essential medicine lists in many LMICs like Malawi. In addition, the few available treatment options, such as cetylpyridinium chloride, amine fluorides, triclosan, and chlorhexidine, are toxic and cause tooth staining^11^. These problems have recently led to the search for alternative oral healthcare medicine and hygiene products from different sources, including herbal medicines. Herbal medicines have become popular in the oral health field and have led to the development of herbal medicine-based products due to the widely held view that they have low toxicity and fewer side effects^12^.

Lately, herbal medicine properties are being exploited for tooth pain, gum inflammation, canker sores management, and bleeding prevention, as well as antimicrobial, antiseptic, antioxidant, and analgesic effects^1–3^. Recently, plant extracts of propolis, noni fruit, burdock root, and neem leaf have been used in periodontics and endodontics as intra-canal medications with excellent results^4,5^. *Acacia cornigera* (L.) Willd has been used for the inflammation of the gums; *Acacia farnesiana* (L.) Willd for cold sores; *Amphipterygium adstringens* Schiede ex Schlech for periodontitis; *Asclepias curassavica* L. for caries and toothache; *Bidens odorata* Cav, *Caesalpinia pulcherrima* (L.), *Carica papaya* L. and *Heterotheca inuloides* Cass for canker sores; *Byrsonima crassifolia* (L.) Kunth, *Capsicum frutescens* L., *Chenopodium graveolens* (Willd.), *Chiranthodendron pentadactylon* and *Heliopsis longipes* (A. Gray) S.F. Blake for toothache; *Jatropha gaumeri* Greenm for oral candidiasis, and tooth abscess; *Opuntia ficus-indica* (L.) Miller for oral ulcer and tooth abscess and *Persea americana* Miller for canker sores, gingivitis, periodontal disease, and toothache. Many medicinal plants have also been tested for plaque accumulation inhibition after oral rinse, inflammation reduction, gingival bleeding reduction, suppression of cell growth, hypo-salivation, and mouth pain amelioration^13,14^. Prevention of deleterious effects by interleukin-1beta in periodontal diseases, suppression of caries development and antibacterial activities of herbal extracts on various bacterial strains (streptococci, *Staphylococcus mutans*, enterobacteriaceae, and *Staphylococcus aureus*) that cause oral infections have also been investigated^15^.

Several medicinal plants have been widely used for many diseases in Malawi^16,17^. However, their effectiveness and possible clinical applications have had little or no scientific study. For example, *Multidentia crassa* (Hiern) Bridson & Verdc of Rubiaceae is widely used for oral health problems such as toothache, but there is no study on its claimed uses and toxicity. *M. crassa* is a shrub with stout branches, leaves mostly restricted to branch apices (3–27.5 cm long), and black or rough grey bark that turns reddish under the surface when peeled. It is mostly found in Brachystegia woodland, or open bushland with scattered trees, forest edges, and rocky outcrops. It is found in many countries such as Angola, Burundi, the DRC, Kenya, Malawi, Mozambique, Sudan, Tanzania, Uganda, Zambia, and Zimbabwe. In Malawi, it is available in all the regions (north, central, and south). This study investigated the chemical composition and therapeutic potential of *M. crassa*. The gas chromatography-mass spectrometry (GC-MS)^18,19^ and fourier transform infrared (FT-IR) spectrometry^20^ were used to identify compounds and functional groups in the extracts of this plant species. Molecular docking was used to evaluate the therapeutic potential of the identified compounds by testing their binding affinities on druggable receptor proteins in dental pain and inflammation pathways that involve activation of classical cyclooxygenases, interleukin-1 beta receptors^21^, odontoblast cold sensor proteins^22^, and the purinergic receptor P2X3^23–30^.

## Materials and methods

**Figure 1:**
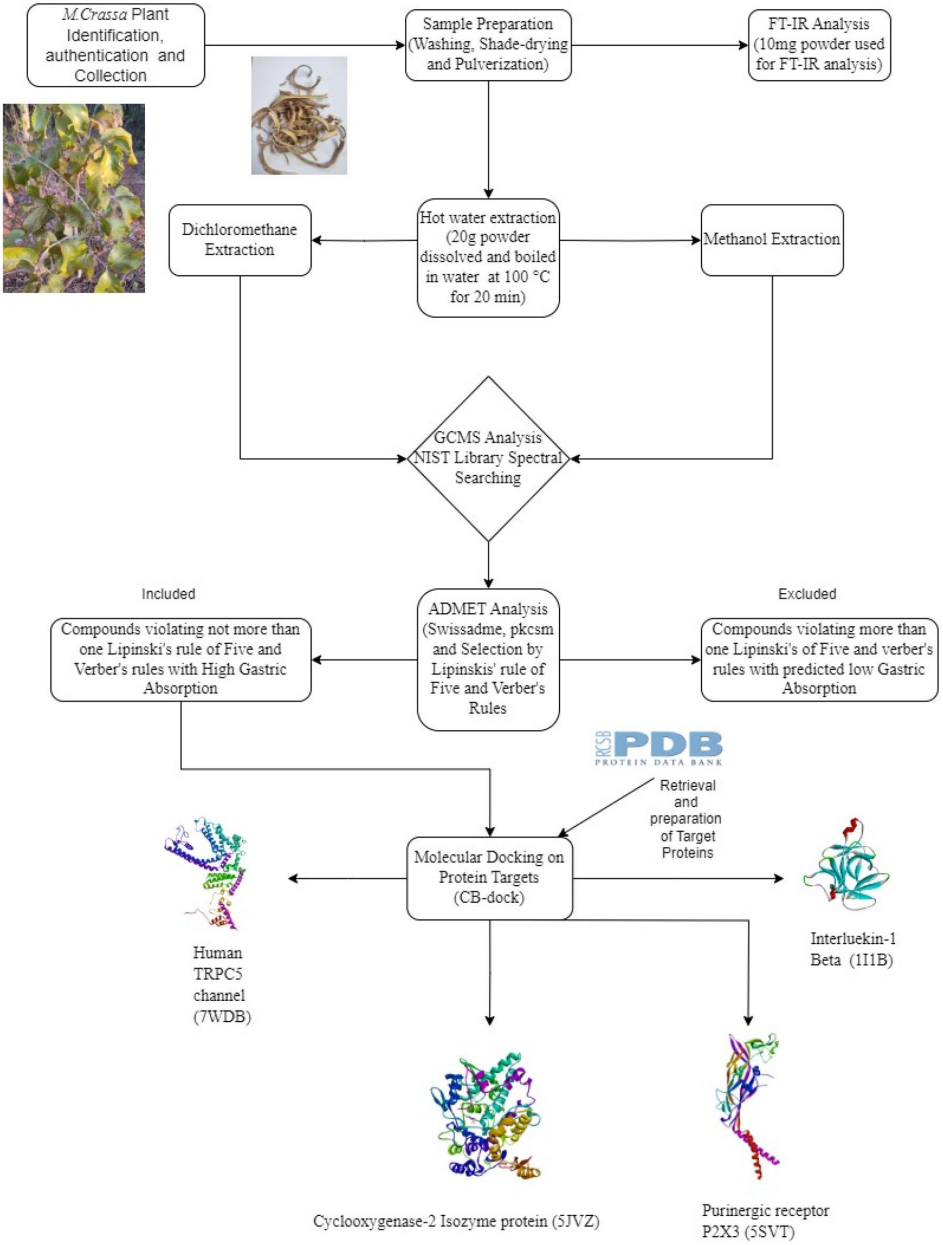
Methodology flowchart for the study.

This study evaluated *M. crassa* using GC–MS, FT-IR, and computational methods. Figure 1 below summarises the steps that were followed in the analyses.

### Collection of plant material

**Figure 2:**
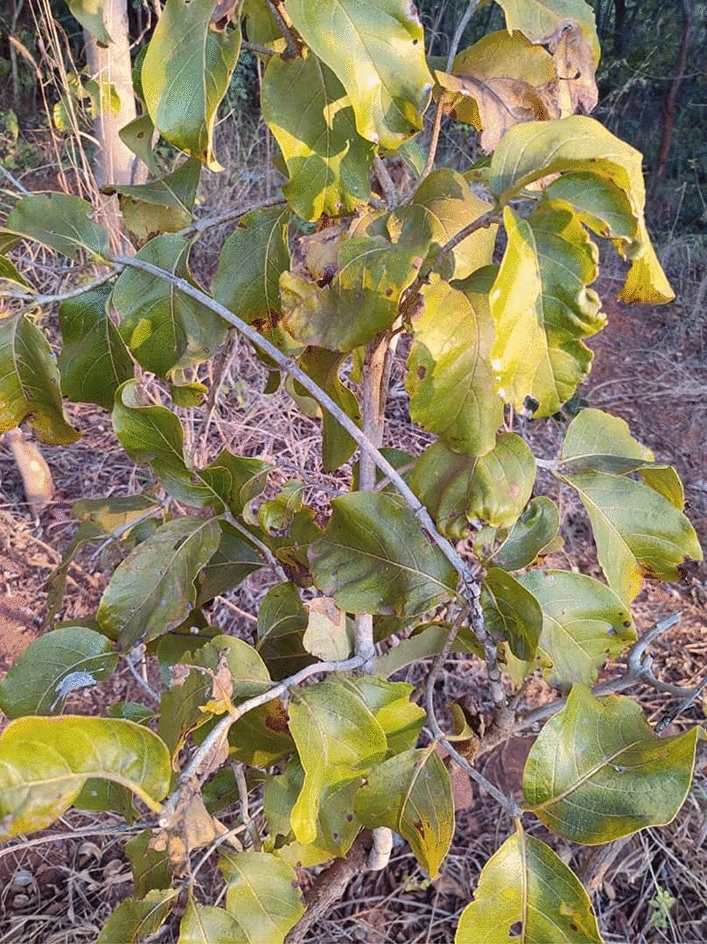
*M. crassa* plant from which the sample was collected.

A sample of *M. crassa* was collected from the Zomba Mountain Forest Reserves, where it is widely available. Identification of the plant was initially done by an herbalist, followed by a literature review, and finally by a botany technician from the Forest Research Institute of Malawi (FRIM). The fresh leaves and bark were collected and taken to the National Herbarium and Botanical Gardens (NHBG) for authentication, and voucher number 0046048 was found to be a match for the plant species. The collection of plant material complied with relevant institutional, national, and international guidelines and legislation. Figure 2 below shows one of the plants where the sample was collected.

### Preparation and extraction of plant materials

The fresh bark of *M. crassa* was washed under running distilled water, shade dried at room temperature (25–30 °C) for 1 week, and pulverized using a food-grade grinder. Ten milligrams (10 mg) of the powder were used in the FT-IR analysis. Twenty grams (20 g) of the powder were soaked in water and boiled for 20 min, mimicking the way it was prepared locally. Since water extracts cannot be analysed in a GC–MS system, the water extracts supernatant was split into two portions and separately mixed with dichloromethane (DCM) and methanol (MeOH). The mixtures were filtered using Whatman filter paper Grade 1 to remove the powder residues, while residual water was removed using a drying agent, anhydrous sodium sulphate. Each solvent mixture was concentrated using a rotary evaporator at 60 °C, followed by filtering and GC–MS analysis.

### Fourier transform infrared (FT-IR) spectrometry analysis

Approximately 10 mg of the plant powder was encapsulated into a pellet by mixing it with half a microspatula of chromatographic-grade potassium bromide (KBr). The powdered sample was then loaded into a Shimadzu Fourier Transform Infrared Spectrophotometer, model IRPrestige21, with a resolution of 4 cm^−1^ and a scanning time of 2–3 s. The instrument uses a deuterated L-alanine doped triglycine sulphate (DLATGS or DLTGS) detector with a scanning range of 7800–350 cm^−1^.

### Gas chromatography-mass spectrometry (GC–MS) analysis

The GC–MS analysis of the sample was done using adapted methods described in the literature^19,20,31–35^. Five milligrams (5 mg) of each herbal extract were added into a Thermo Scientific Reacti-Vial containing a Reacti-Vial magnetic stirrer. MeOH and DCM solvents were added separately to each vial, stirred, and filtered using MS microfilters. The filtrate was transferred to a 2 mL autosampler vial. One (1) μL of each sample was injected into the Agilent Technologies 5975C Inert XL EI/CI MSD with Triple-Axis Detector GC–MS. A gas chromatography column of specifications J and J Scientific Part Number: 122-5012, DB-5 GC column, 15 m × 0.25 mm × 0.25 μm, 7-inch cage Agilent Technologies 5975c was used for the analyses. The extracts were separated at a programmed oven temperature as follows: Oven temperature: 40 °C (1 min), 10 °C/min up to 260 °C, 260 °C (5 min). The injector temperature was set at 200 °C, while the detector temperature was 250 °C. Helium (He) was a carrier gas at a constant flow of 1.2 mL/min. An autosampler injection volume of 1 μL was injected with a split flow of 60 mL/min. The system was operated with a detector hydrogen flow of 35 mL/min, a detector air flow of 350 mL/min, and a detector nitrogen flow of 30 mL/min. The mass spectrum was referred to a computer-fed mass spectra data bank. The separated components were translated into mass spectra peaks and identified by comparing them with mass spectra chromatograms in the built-in National Institute of Standards and Technology’s (NIST) GC–MS library software. The NIST uses the submitted unknown spectrum from mass spectrometer detectors (MSD) and performs a library spectrum search. Based on the confidence level, a hit list of compounds matching chemical structures of the sample compounds is produced. A NIST library probability score of at least 50% matching in GC-MS analysis was used to select a compound from the hit list. The NIST library probability score is a measure of similarity between a compound in the sample given in a peak in a GC-MS analysis and known compounds in the NIST library^36^. The functional groups in the identified compounds from the GC-MS analysis were compared with the functional groups identified in the FT-IR analysis.

## Active compounds (ligands) retrieval and preparation

The GC-MS-identified bioactive compounds were searched on PubChem (http://pubchem.ncbi.nlm.nih.gov/) and the NIST library (https://webbook.nist.gov/chemistry/cas-ser/) to retrieve their 2D/3D structures, and saved in a structure data file (*.sdf file) format. Canonical simplified molecular-input line-entry system (SMILES) names of the bioactive compounds were retrieved from the same sources and used to retrieve the test compound structures in software for pharmacokinetic (absorption, distribution, metabolism, excretion), toxicity, and pharmacodynamic (compound–target binding) tests. The physicochemical properties of the compounds were collected from the PubChem database, and compounds without a PubChem compound identification number (CID) were excluded.

### In silico pharmacokinetic and toxicity analysis of phytocompounds

Structures of the selected compounds saved in ‘.sdf’ format with their corresponding canonical SMILES were evaluated using the SwissADME (http://www.swissadme.ch/)^35^ and pkCSM (https://biosig.lab.uq.edu.au/pkcsm/prediction) online web servers^37–39^. The data from these computational tools were used to evaluate and predict the physicochemical properties, pharmacokinetics, and medicinal chemistry friendliness of phytocompounds. The information collected and evaluated from the tools included molecular weight, lipophilicity, or hydrophilicity, gastrointestinal absorption, blood–brain barrier (BBB) permeability, p-glycoprotein substrate, skin permeability, toxicity, drug likeliness, and bioavailability score, among others^35^. Drug-likeness is demonstrated by aqueous solubility represented by parameters log S in estimating aqueous solubility (ESOL) model, oral bioavailability demonstrated by Veber classification or rule (number of rotatable bond count;< 10), and topological polar surface area (TPSA); < 140 Å^2^) and Lipinski’s rule of five, i.e., the molecular weight of not more than 500 g/mol; at most 5 hydrogen bond donors (HBDs); at most 10 hydrogen bond acceptors (HBAs); and calculated octanol–water partition coefficient (C log P) values less than 5; and compounds must not violate more than 1 rule of Lipinski’s rule of five. The molecular weight determines the density, size, and volume of a compound while the HBA and HBD determine membrane transport, drug-protein interactions, distribution, and aqueous solubility. A compound is not suitable for drug development studies if it violates some of the rules prescribed in each model^37,40^.

Toxicity evaluation was done using the following virtual assays: Ames toxicity that uses modified bacteria sensitive to mutagenic agents data to assess the ability of a compound to cause direct DNA mutations; hERG I/II inhibition (the capacity of a compound to inhibit the human ether-à-go-go-related gene (hERG) cardiac potassium channel, causing cardiotoxicity); oral rat acute toxicity (LD50), which is a statistically derived dose that causes death in 50% of the treated animals in a given period; oral rat chronic toxicity lowest observed adverse effect level (LOAEL), the lowest exposure level at which there are biologically significant increases in the severity of adverse effects; *Tetrahymena pyriformis* toxicity, the concentration of a compound that inhibits growth of 50% *Tetrahymena pyriformis* (IGC50); and Minnow toxicity (lethal concentration, LC50, that causes the death of 50% fathead minnows larvae). Hepatotoxicity and skin sensitization were also evaluated.

### Comparative analysis of pkCSM and SwissADME results

The pkCSM and SwissADME results were compared to determine the level of agreement between the two ADME analytical tools. Measurement units were standardised and uncommon parameters between the two tools were excluded. The comparison data were represented by a frequency table (for categorical variables) and a Bland–Altman plot (for continuous variables). The proportions or percentages, and Cohen’s kappa statistical evaluations were performed to assess the level of agreement between the tools^41^.

### In silico pharmacodynamic analysis of phytocompounds Protein identification and preparation

**Figure 3:**
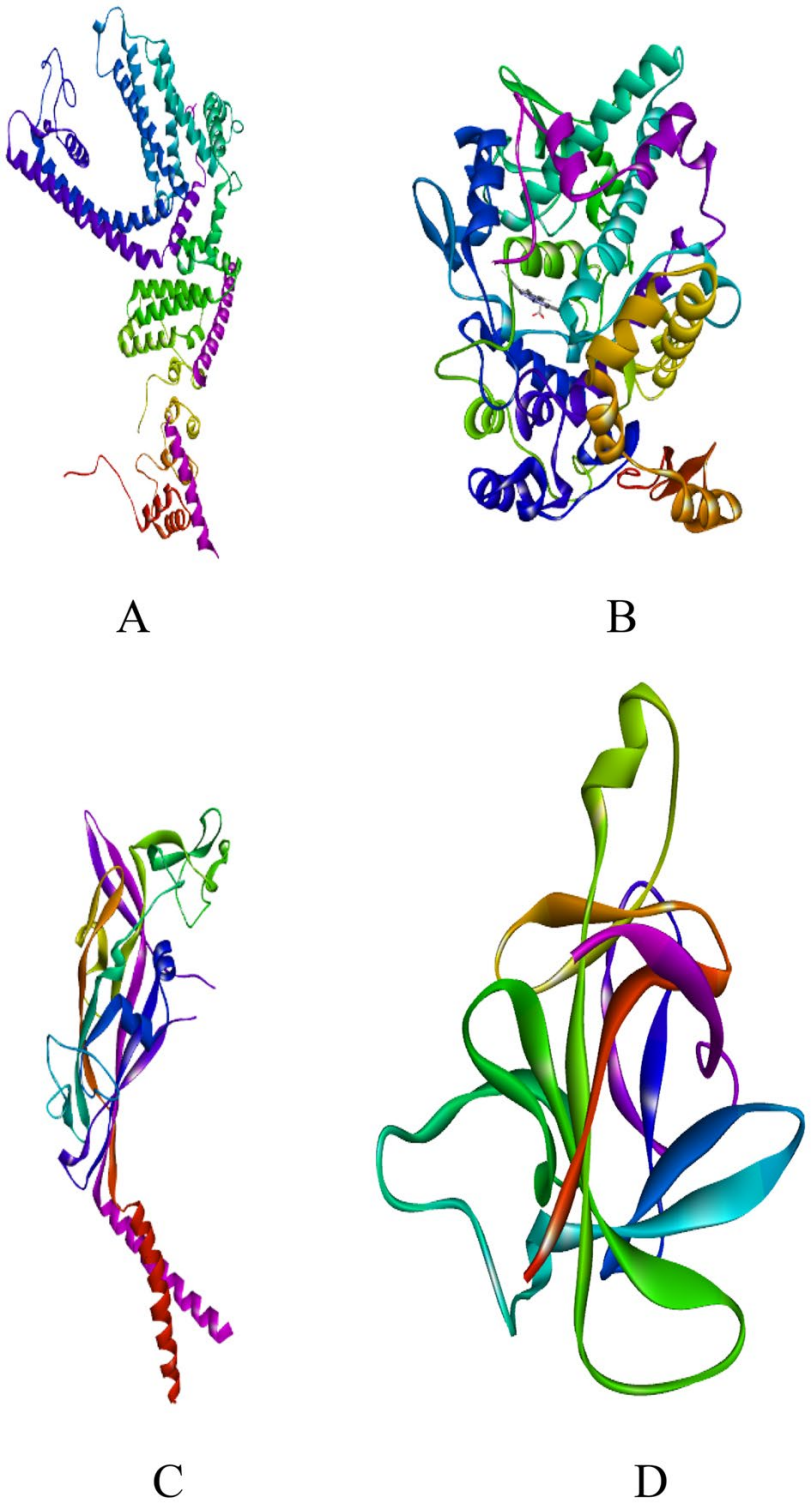
X-ray crystallographic structures (**A**–**D**) of targeted proteins as drug targets. (**A**) human TRPC5, (**B**) cyclooxygenase 2, (**C**) purinergic receptor P2X3 and (**D**) interleukin-1 beta receptor.

Molecular docking was used to study some of the pharmacodynamic properties of the phytocompounds. One of the uses of molecular docking is the prediction of the binding of ligands (small molecules) to proteins. Pharmacodynamic analyses were done on the prepared protein targets associated with oral health or dental diseases, particularly anti-inflammatory, analgesic, and thermosensitivity activities. These included four selected target proteins, namely odontoblast cold sensor protein (TRPC5) (protein bank reference number 7WDB)^22^, cyclooxygenase-2 isozyme protein (protein bank reference number 5JVZ)^42^, purinergic receptor P2X3^43,44^, and interleukin-1 beta (protein bank reference number 1ITB)^45,46^. The X-ray crystallographic structures of these target proteins (Fig. 3) were obtained from the RCSB Protein Data Bank (https://www.rcsb.org/pdb). Water molecules and bound ligands were deleted while missing residues and atoms were repaired and inserted respectively, and the polar hydrogens were added to the protein using Autodock Vina software. The water molecules were deleted because they increased the complexity of the molecular docking. Water molecules also increase the computational cost by introducing noise and incorrect binding poses. Hence, the water molecules deletion cleared the active site for easy ligand fitting into the active site cavity (which would be impossible in the presence of water molecules or other ligands). In addition, Kollman partial charges were introduced and spread throughout the overall structure.

### Molecular docking

The GC-MS-identified  phytocompounds were evaluated further for selection into molecular docking using Lipinski’s rule of five drug-likeness tests^36^. The selected compounds were subjected to binding interactions with cold sensor protein (TRPC5), cyclooxygenase-2 isozyme protein, purinergic receptor P2X3, and interleukin-1 beta (Fig. 3). To compute docking energy affinities (kcal mol^−1^), the receptor and ligand files in .pdb and .mol formats, respectively, were run on the CB-Dock web server (http://clab.labshare.cn/cb-dock)^47^. The CB-Dock is a protein–ligand docking tool that automatically identifies the binding sites (cavities), calculates the centre and size, customizes the docking box size of the protein drug target according to the query ligands, and then performs the molecular docking with AutoDock Vina. For each ligand, AutoDock Vina calculates the energy affinity values of up to five different docking positions. AutoDock Vina calculates protein–ligand complex affinity energy based on the ligand conformations at the active binding site, and the root mean square deviation (RMSD) of atomic positions between the original and subsequent structures is considered. The protein–ligand complex with the lowest binding energy (highest binding affinity) for each compound was selected to identify amino acid residues involved in the binding, the bond type and the bond length.

### Literature review on traditional uses, bioactivity, and toxicity data of the plant and the identified phytocompounds

The GC-MS-identified phytocompounds were also searched in the literature particularly Google Scholar and PubMed search engines to find out previously reported bioactivity and toxicity data and similar studies about the plant.

### Statistical analysis

All experiments were done in duplicate, and the mean results were reported. All graphs were plotted using Origin 2023 and Microsoft Excel 2021.

## Results

### Fourier transform infrared (FT-IR) spectrometry analysis

**Figure 4:**
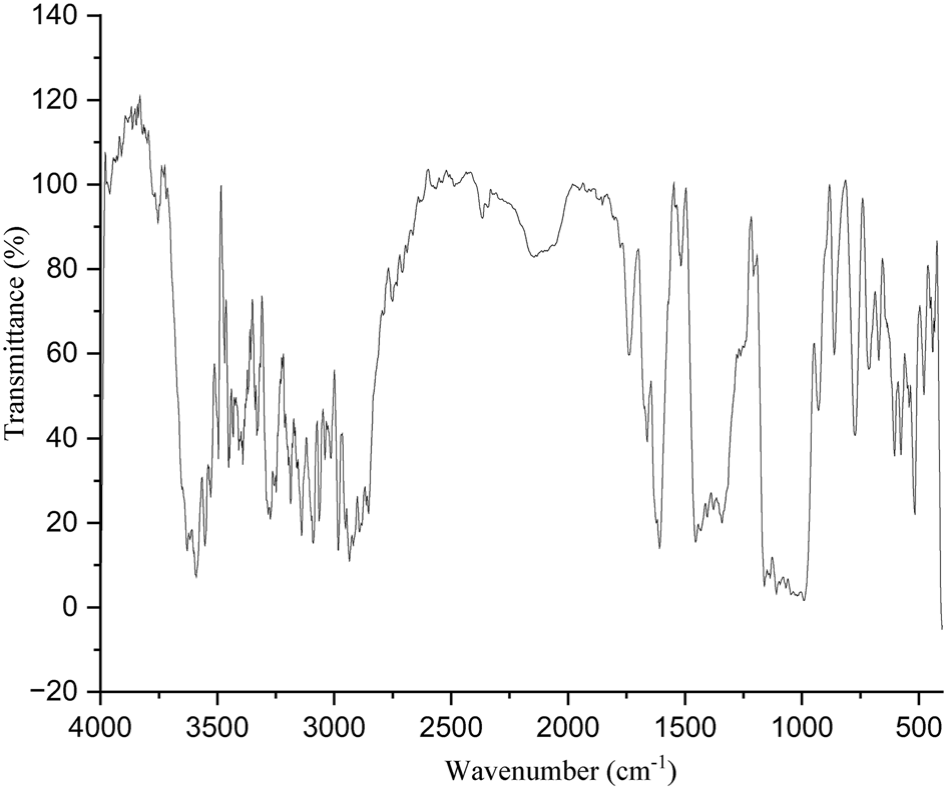
FT-IR spectrum for *M. crassa.*

FT-IR spectroscopy was used to identify functional groups of bioactive compounds in the powder of *M. crassa*. Absorption bands were observed between 432 and 3718 cm^−1^ (Fig. 4 and Table 1). The results showed that the plant extract contained hydroxyl groups (O–H) from alcohols, carboxylic acids, and phenols; carbonyls (C=O) from aldehydes, carboxylic acids, esters and amides as well as alkanes (C–C), alkenes (C=C), amines (N–H), alkynes (C≡C), haloalkane compounds, ethers, and aromatics . These functional groups arise from many compounds from various classes of phytochemicals such as alkaloids, flavonoids, terpenoids, polyphenols, and tannins^48^.

**Table 1:** FT-IR data of bark of *M. crassa.*

Frequency (cm^−1^)	Types of compounds	Bond responsible	GC–MS compounds
432–671	Cyclohexanes, cis-alkenes, alkynes, aromatic aliphatic nitrile, azide, phenols, aromatics, aliphatic ethers, primary aromatic amines, secondary aliphatic amines, saturated aliphatic chlorides, aliphatic chloroformate, amide, benzene, 4-substituted pyrimidines	Not mentioned	Cyclohexanes, cis-alkenes, alkynes, aromatics, phenols, aromatics, aliphatic ethers, amines, saturated aliphatic chlorides, amide, benzene
713–991	Alkene	C=C	Alkene
1014	Ether, alkanol	C–O	Ether
1020–1275	Alkene, ether	C–H	Alkene
1069–1144	Ether, alkanol	C–O	Ether, alkanol
1047–1144	Alkanol	COH	Alkanol
1047–1069	S=O	S=O	Absent
1028–1144	P-O-Alkyl	P–O	Absent
1028–1109	Aromatics	C–Cl/Br	Alkyl halide or bromine or chlorine
1207	Ester	C–O	Ester
1275–1454	Cyclic propane, acetates, sec alkanol, amines,	CH deformation vibrations	Alkane, alkanol, amine
1342–1661	Alkene	C=C stretching vibration	Alkene
1518	Polyglycines	NH deformation vibrations	Absent
1454–1661	Imine, quinone oximes, Polyglycines, aliphatic azoxy compounds, sec amines, amino acids, Beta diketones (metal chelates), amides, sec urethanes	C=N stretching vibration, NH deformation vibrations, NH bending vibrations, NH^3+^ deformation vibrations, CHN	Amines, amides
1740–1842	Ester carbonyl	C=O stretching vibration	Ester
1802–1854	Vinyl hydrocarbon compounds	CH vibration	Alkene
1776–1854	Ketones	C=O stretching vibrations	Absent
1802–1919	Sn–H	Stretching vibrations	Absent
1842–1919	Amino acids	Lys side chain, amide, or peptide bond of the amino acids	Absent
1802–2853	Acetals	CH stretching	Absent
2511–3528	Quinone oximes, chelated OH, intramolecular bonded	OH stretching vibration	Absent
2053–2951	aromatic, cyclic, and acyclic compounds and aldehydes	CH stretching vibrations and deformations overtones	Aromatics, aldehyde, cyclic alkanes/alkene, and acyclic alkanes/alkenes
2750–2951	Aromatic methylene dioxy compounds	CH stretching vibration	Absent
2862–2918	Ethers and RSCH_3_	C–H	Ether
2853–3316	Amines and alkanols	NH and OH stretching vibration	Amine, alkanol
3098	Carboxylic acid	OH	Carboxylic acid
3157–3211	Alkynes	CH	Alkyne
3211–3628	Oximes, free OH, silanos, kaolin	OH-vibration	Alkanol
3356	Alcohol	OH	Alkanol

### Gas chromatography-mass spectrometry (GC–MS) analysis

**Figure 5:**
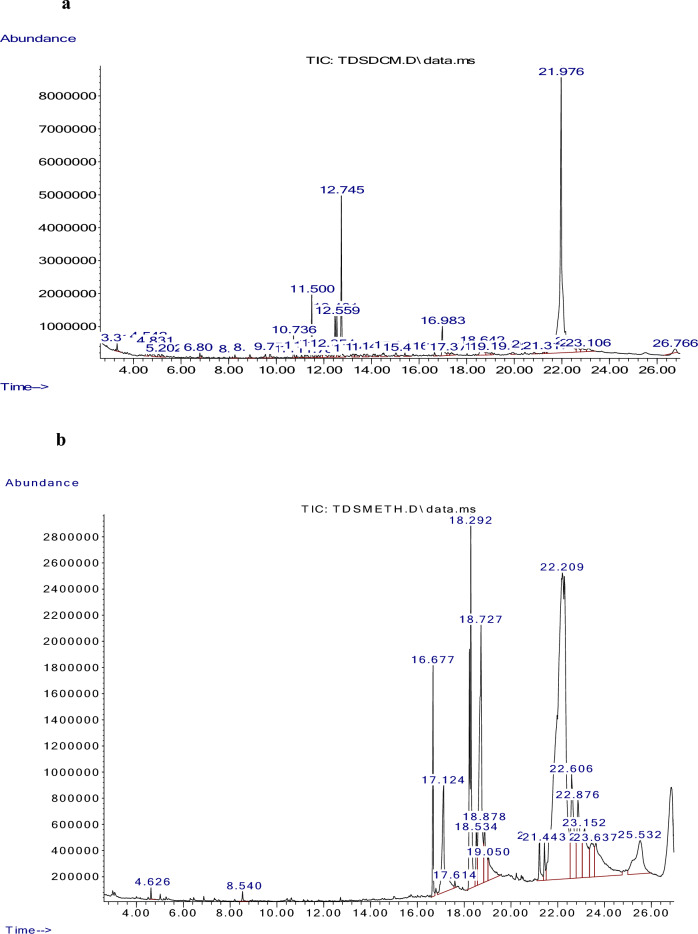
(**a**) GC-MS chromatogram for the DCM extract of *M. crassa*. (**b**) GC–MS chromatogram for the methanol extract of *M. crassa.*

The GC–MS analysis showed that the plant extracts had over 58 compounds  (46 DCM extract compounds and 12 methanol extract compounds). Figures 5a and b show the DCM and methanol chromatograms respectively while Additional File: Table 1 shows the details of the identified compounds.

### GC–MS-identified compounds and their bioactivities

The GC–MS showed that the plant extracts had many compounds and a literature review showed that some of the compounds had both pharmacological and toxicity studies. For example, some of the DCM extract compounds reported in the literature had topical and systemic anti-inflammatory, anticancer, antioxidant, anti-influenza, antimicrobial, anti-shigellosis, pro-inflammatory, antimutagenic, cytotoxic, analgesic, antipyretic, down-regulation of protein expression of iNOS and COX-2, and antiproliferative (against HeLa cells) activities. On the other hand, some compounds from methanol extract reported in the literature had anti-inflammatory, antiandrogenic, dermatitigenic, hypocholesterolemic, anemiagenic, insectifuge, antioxidant, cancer prevention, hepatoprotective, nematicide, antihistaminic, antieczemic, antiacne, alpha-reductase inhibitor, anti-coronary, and antimicrobial activities (Additional File: Table 1)^31,33,34,49–70^. Some of the bioactivities reported in the literature were directly related to oral health such as anti-inflammatory, analgesic, and antipyretic activities^2,3^. A review of the literature about the plant showed that there was no report on GC-MS-based plant metabolic characterization of its bioactive compounds. Therefore, the study has generated baseline data for further studies of the plant.

### In silico testing of the molecular properties, pharmacokinetics, and toxicity of GC–MS-identified compounds

#### In silico molecular properties of the identified compounds

This involved the analysis of the drug-likeness of the *M. crassa* phytocompounds using pkCSM and SwissADME-generated data. The data from these online tools included absorption, distribution, metabolism, and excretion (ADME) properties. All compounds (100%) from both MeOH and DCM extracts passed the drug-likeness test of Lipinski’s rule of five since none of them had violated more than one of its requirements. However, 74% (43/58) of the compounds passed the Veber classifications. In addition, 74% (43/58) of the compounds passed both Lipinski’s rule of five and Veber's classification (Additional File: Table 2).

#### In silico pharmacokinetic parameters of the identified compounds

Oral absorption, distribution, metabolism, and excretion (ADME) of the GC–MS-identified compounds were tested using the pkCSM and SwissADME platforms (Additional File: Tables 3, 4, 5, and 6). Absorption was predicted using water solubility, lipophilicity, and percentage of human intestinal absorption (HIA) modeling on the pkCSM platform. In SwissADME, water solubility and/or absorption were determined using Silicos IT and Ali solubility parameters (Additional File: Table 5). The HIA percentages, which show the amount of the compounds absorbed in the intestines, were very high, falling between 87.68 and 96.67% for all compounds (Additional File: Table 3a). Lipophilicity measures the ability of a substance to dissolve in lipids or fats and affects its pharmacokinetic profile. For example, high lipophilicity (Log P > 5) causes high metabolism, low solubility of substance, and poor oral absorption. The optimal physicochemical property of ADME is achieved when log P is between 1 and 5^71,72^. This is measured using various methods including skin permeability (log Kp) and partition coefficient (log P). Twenty-nine percent (29%, 17/58) of the compounds had high skin permeability (log Kp > − 2.5), and 59% (10/17) of these were from methanol extract. 2-Amino-3,5-dibromopyridine had the lowest log Kp (− 3.109) (highest permeability) and (-)-cis-beta-elemene had the highest log Kp (− 1.279) (lowest permeability). These results were like the SwissADME results. P-glycoprotein substrate and inhibition status of the compounds were also predicted and four compounds were p-gp substrates, three compounds were p-gp I inhibitors, and nine compounds were p-gp II inhibitors.

The distribution of the compounds in the body was measured using the volume of distribution in humans (VDss), plasma protein binding (fraction unbound in humans, Fu), and passage through the blood–brain barrier (BBB) and central nervous system (CNS). For VDss, only 9% (5/58) of the compounds namely 9-octadecenoic acid, elaidic acid, oleic acid, palmitic acid, and phthalic acid, di(2-propylphenyl) ester, had a low volume of distribution (< − 0.15), while the rest had medium to high VDss (low if logVDss < − 0.15 and high if logVDss > 0.45)^39^. Twelve percent (12%, 7/58) of the compounds had zero fraction unbound (Fu) and these were eicosane, octadecane, stigmastan-3,5-diene, diisooctyl phthalate, bis(2-ethylhexyl) phthalate, 1,4-di-tert-butylbenzene, and 1,3-di-tert-butylbenzene. Based on the pkCSM rules, 78% (45/58) of the compounds could penetrate the BBB, while 72% (42/58) could penetrate the CNS barrier. The SwissADME platform showed that 31% (18/58) of the compounds could cross the BBB. Based on the results above, some compounds had favourable distribution properties for druggable compounds.

In metabolism, none of the compounds were CYP2D6 substrates or inhibitors. But half of the compounds (50%, 29/58) and 2% (1/58) were CYP3A4 substrates and inhibitors, respectively, and the lone CYP3A4 inhibitor was phthalic acid, di(2-propylphenyl) ester. Thirty-six percent (36%, 21/58) of the compounds were CYP1A2 isozyme inhibitors, while 10% (6/58) and 7% (4/58) were CYP2C19 and CYP2C9 inhibitors, respectively.

In excretion, renal clearance and renal substrate activity were studied. Renal clearance was evaluated by classifying the compounds into three categories: high (> 1 mL/min/kg), medium (> 0.1 to < 1 mL/min/kg), or low (≤ 0.1 mL/min/kg). Sixty-two percent (62%, 36/58) of the compounds had high renal clearance, and eicosane had the highest renal clearance (2 mL/min/kg), 36% (21/58) had medium renal clearance, 2% (1/58) had low renal clearance, and 2-amino-3,5-dibromopyridine had the lowest renal clearance (− 0.14 mL/min/kg). Since the compounds had renal clearance, they were also evaluated for their potential as OCT2 substrates (an important renal transporter) and inhibitors, as recommended by the Food and Drug Authority (FDA) and European Medicines Authority (EMA) for compounds with renal clearance. The results showed that only one compound, (1E,5E,11E)-1,5,11-trimethyl-8-isopropenylcyclotetradeca-1,5,11-triene) was an OCT2 substrate (Additional File: Tables 3 and 4).

#### In silico toxicity prediction of the identified compounds

The following parameters were predicted for the toxicity profiles of the phytocompounds: Ames toxicity, maximum tolerated dose (human), hERG I inhibitors, and hERG II inhibitors, oral rat acute toxicity (LD50), oral rat chronic toxicity lowest observed adverse effect level (LOAEL), hepatotoxicity, skin sensitization, *T. pyriformis* toxicity, and Minnow toxicity. For Ames toxicity, only 1,3-dichloropropane was positive. The maximum recommended toxic dose was low [< 0.477 (log mg/kg/day)] and hence unfavourable for 74% (43/58) of the compounds^73^. All compounds were non-inhibitors of hERG 1, while only 10% (6/58) were hERG II inhibitors. On the other hand, only 3% (2/58) of the compounds namely, phthalic acid, di(2-propylphenyl) ester, and 5-indanol, were hepatotoxic. Approximately 76% (44/58) of the compounds were skin-sensitive. Based on *T. pyriformis* toxicity predictions, all compounds were toxic (> − 0.5 log ug/L). Finally, Minnow toxicity evaluations showed that 29% (17/58) of the compounds were toxic (< − 0.3 log mM) (Additional File: Table 4). Therefore, the results showed that some of the compounds could be toxic.

### Comparative analysis of the pkCSM and SwissADME results

The pkCSM and SwissADME ADMET results were evaluated for consistency. The results showed that only intestinal absorption, distribution, and metabolism were comparable, and their consistencies were 100%, 31%, and 44–95% respectively (Additional File: Table 6).

**Figure 6:**
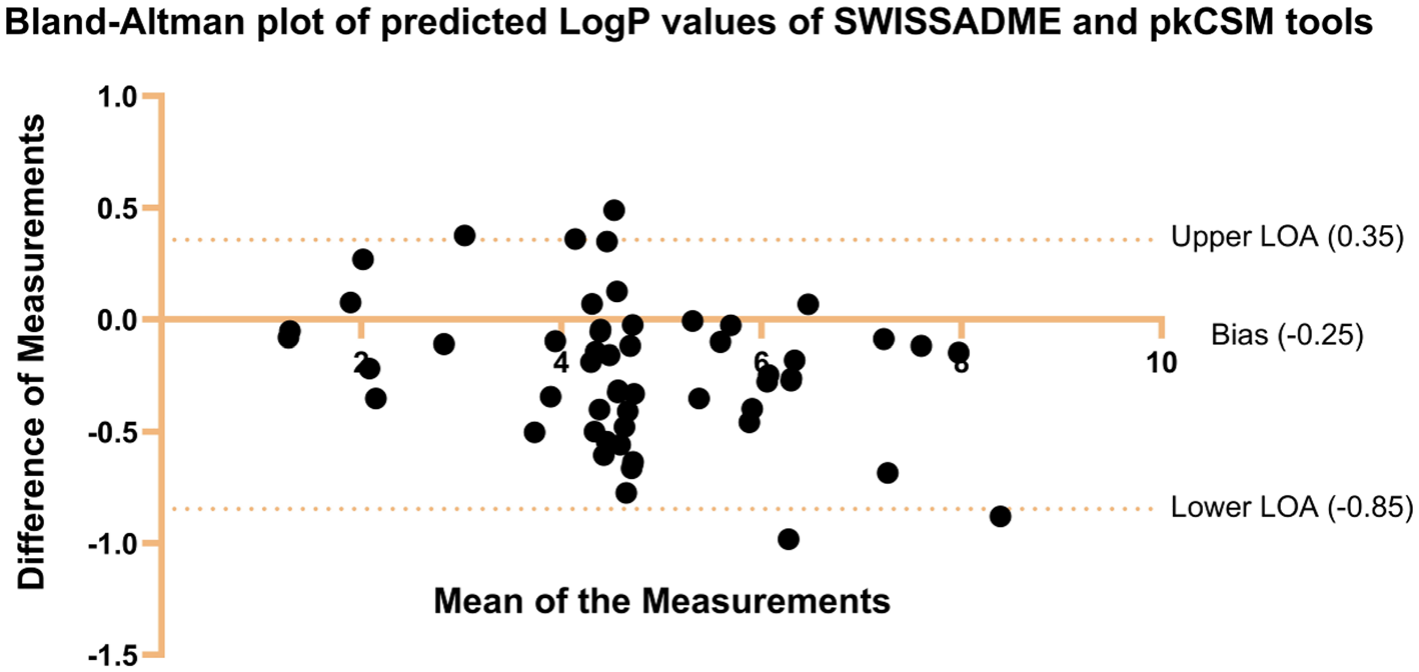
Bland-Altman plot of log P values of SwissADME and pkCSM tools.

The Bland–Altman plot in Figure 6 shows the comparison of the log P measurements from the SwissADME and pkCSM tools. The measurements have a bias of − 0.25 ± 0.3, and 95% limits of agreements (LOA) of − 0.84 and 0.36 (lower and upper LOA respectively). Since the plot follows an irregular pattern and 95% of the points fall within the 95% limit of agreement, the log P measurements from these tools were equivalent.

Agreement between the tools on the categorical data regarding metabolism was calculated using the proportion of agreement and Cohen’s kappa. The following parameters were analysed: intestinal absorption, BBB permeant, P-gp substrate, and CYP450 inhibitors (1A2, 2C19, 2C9, 2D6, and 3A4). The results of the proportion (percentage) of agreement and Cohen’s kappa as measures of agreement between the methods are presented in Table 2. By percentage agreement, intestinal absorption showed the highest proportion of agreement (100%), followed by CYP3A4 inhibitions with a proportion of agreement (95%), while BBB permeation had the lowest percentage of agreement (32%). Cohen’s kappa (calculated at 95% confidence), indicated that the results of the following parameters did not agree: BBB permeant (kappa, CI at 95%) (− 0.11, CI − 0.13 to − 0.10), CYP2C19 inhibitors (− 0.05, CI − 0.09 to − 0.009), and CYP2C9 inhibitors (0.06, CI 0.04 to 0.09). The latter might be due to a lack of comparable parameters (Additional File: Table 6).

**Table 2:** Measures of agreements between SwissADME and pkCSM tools.

S.no.	Percentage of agreement (percentage)	Cohen’s Kappa (95% confidence interval)	Interpretation of Kappa
Intestinal absorption	100%	1	Strong agreement
BBB permeant	31%	− 0.11 (− 0.13 to − 0.10)	No agreement
Pgp substrate	92%	0.2 (0.05 to 0.45)	Slight agreement
CYP1A2 inhibitor	79%	0.53 (0.51 to 0.56)	Moderate agreement
CYP2C19 inhibitor	52%	− 0.05 (− 0.09 to − 0.009)	No agreement
CYP2C9 inhibitor	44%	0.06 (0.04 to 0.09)	No agreement
CYP2D6 inhibitor	93%	0.31 (0.10 to 0.53)	Fair agreement
CYP3A4 inhibitor	95%	0 (− 0.62 to 0.62)	No agreement

### Molecular docking

The molecular docking approach is a crucial component of structural biology research, and it is one of the most widely used techniques in drug design. The top ten (10) compounds from the DCM extract that satisfied Lipinski’s rule of five were selected, while all compounds from the methanol extract were included in the molecular docking study. Four selected target proteins were cold sensor, cyclooxygenase-s, purinergic receptor P2X3, and interleukin-1 beta, which are involved in the analgesic and anti-inflammatory pathways^22,74–76^. The target-compound interactions were found using docking methods and tools properly shown by the negative values of binding energy and/or affinities as well as ligand root-mean-square deviation (RMSD) values of less than 2.0A^77^. The lowest binding energy (highest binding affinity) against the cold sensor was − 10.1 kcal/mol by stigmastan-3,5-diene, while the lowest binding energy was − 3.6 kcal/mol by propane, 1,3-dichloro-. The lowest binding energy (highest binding affinity) against cyclooxygenase-2 was -9.7 kcal/mol by stigmastan-3,5-diene and the highest binding energy (lowest binding affinity) was − 3.8 kcal/mol by propane, 1,2-dichloro. In the interleukin-1 beta receptor, the lowest binding energy (highest binding affinity) (− 7.9 kcal/mol) was achieved by stigmastan-3,5-diene, and the highest binding energy (lowest binding affinity) was− 3.1 kcal/mol by propane, 1,3-dichloro-. Finally, in the purinergic P2X3 receptor, the lowest binding energy (highest binding affinity) was − 6.3 kcal/mol by 7R,8R-8-hydroxy-4-isopropylidene-7-methylbicyclo [5.3.1] undec-1-ene and highest binding energy (lowest binding affinity) was − 3.1 kcal/mol by propane, 1,3-dichloro (Additional File: Table 6).

**Figure 7:**
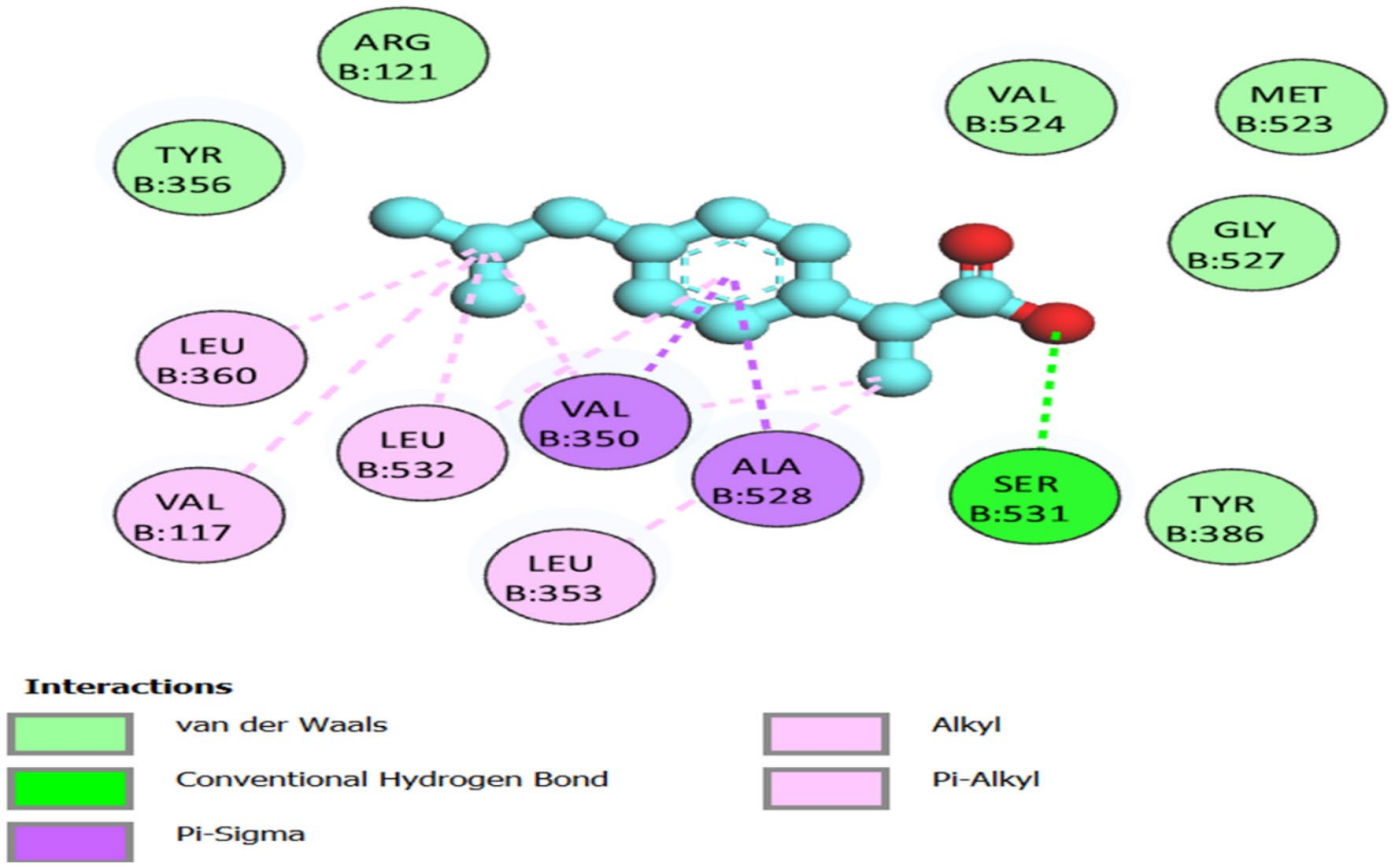
Two-dimensional molecular interactions of re-docked ibuprofen with cyclooxygenase-2 residues.

**Figure 8:**
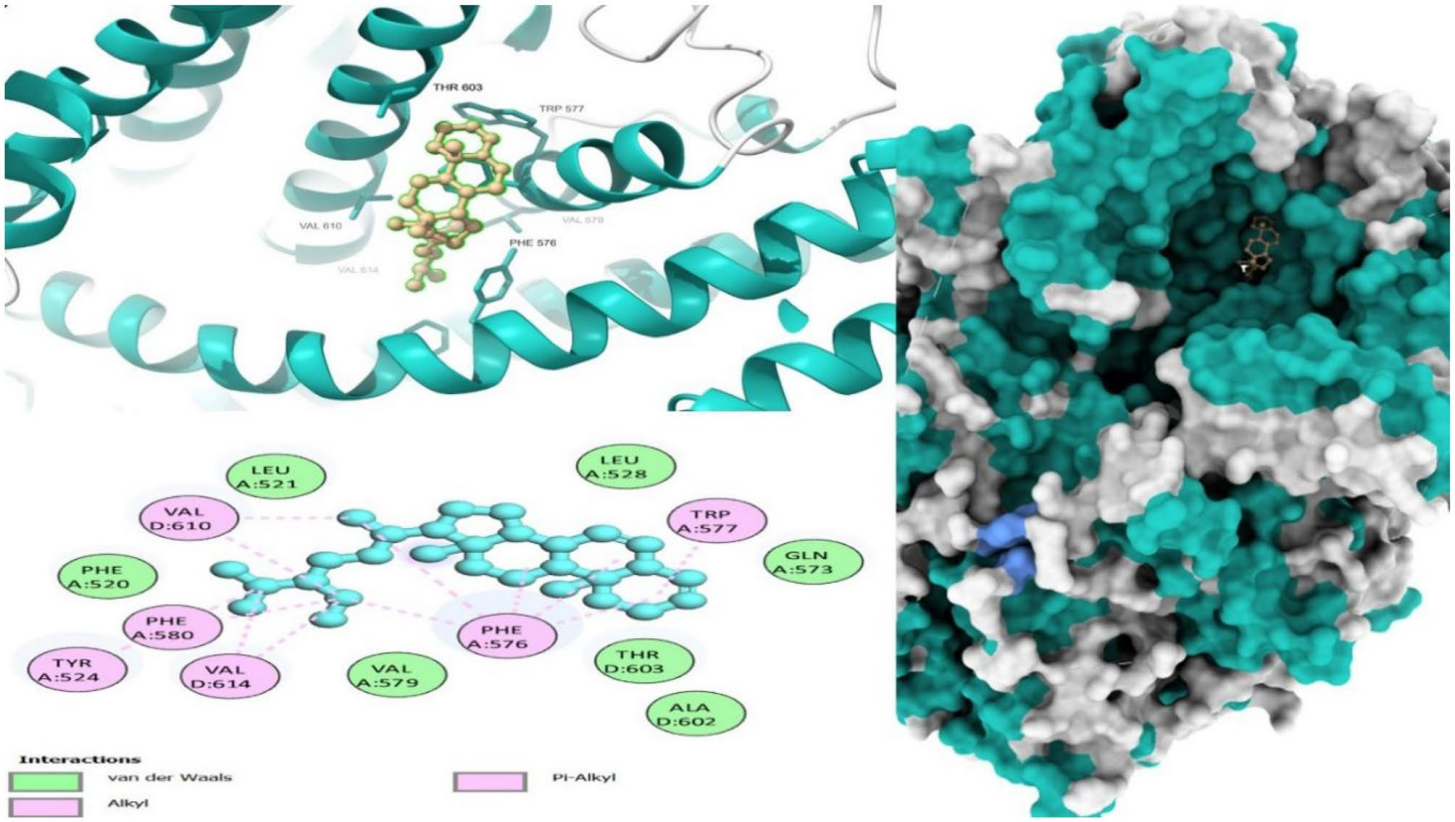
3D, 2D, and mapping surface showing binding modes between stigmastan-3,5-diene and active site residues of cold sensor protein.

**Figure 9:**
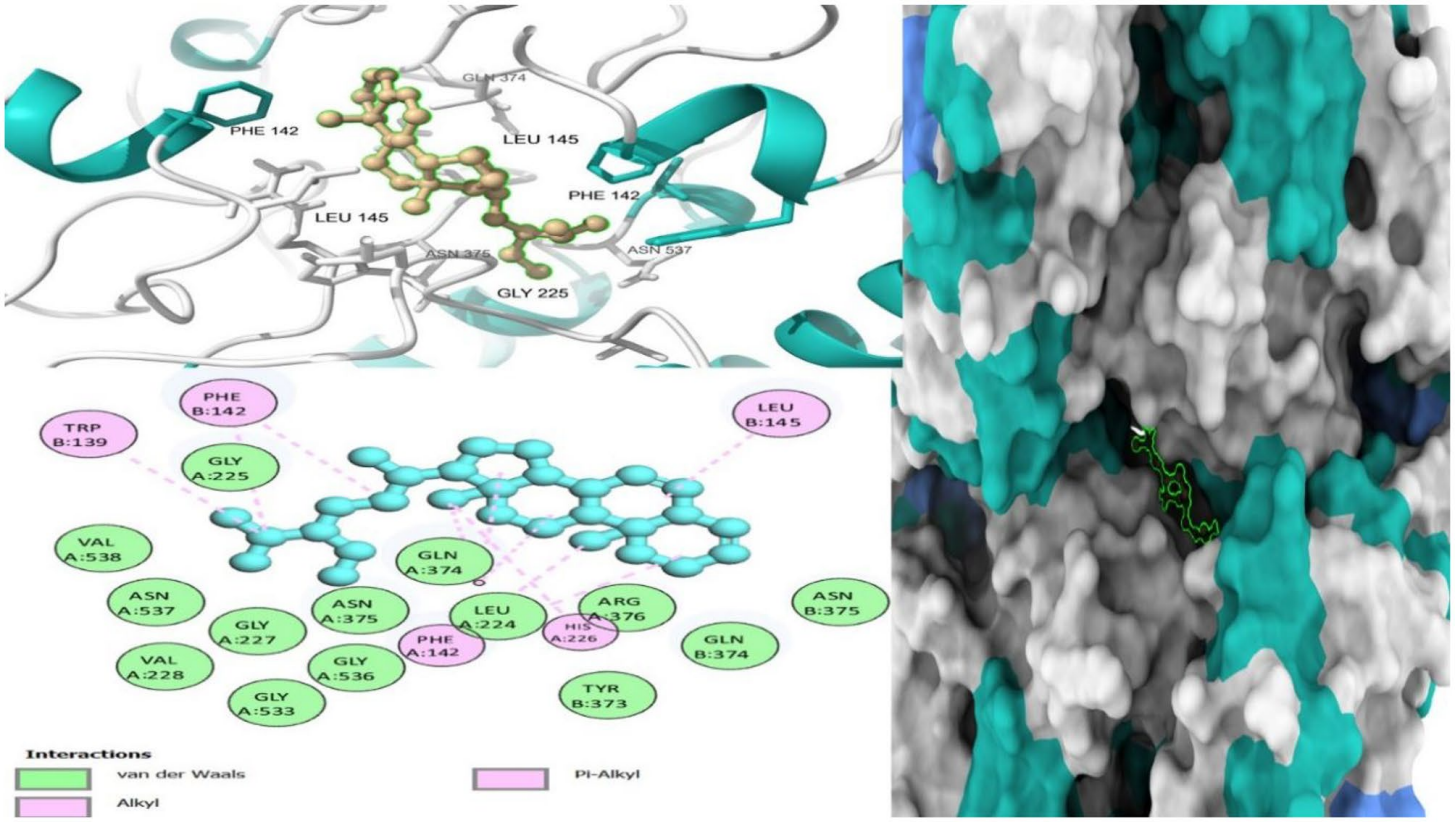
3D, 2D and mapping surface showing binding modes between stigmastan-3,5-diene, and active site residues of *cyclooxyegnase-2.*

**Figure 10:**
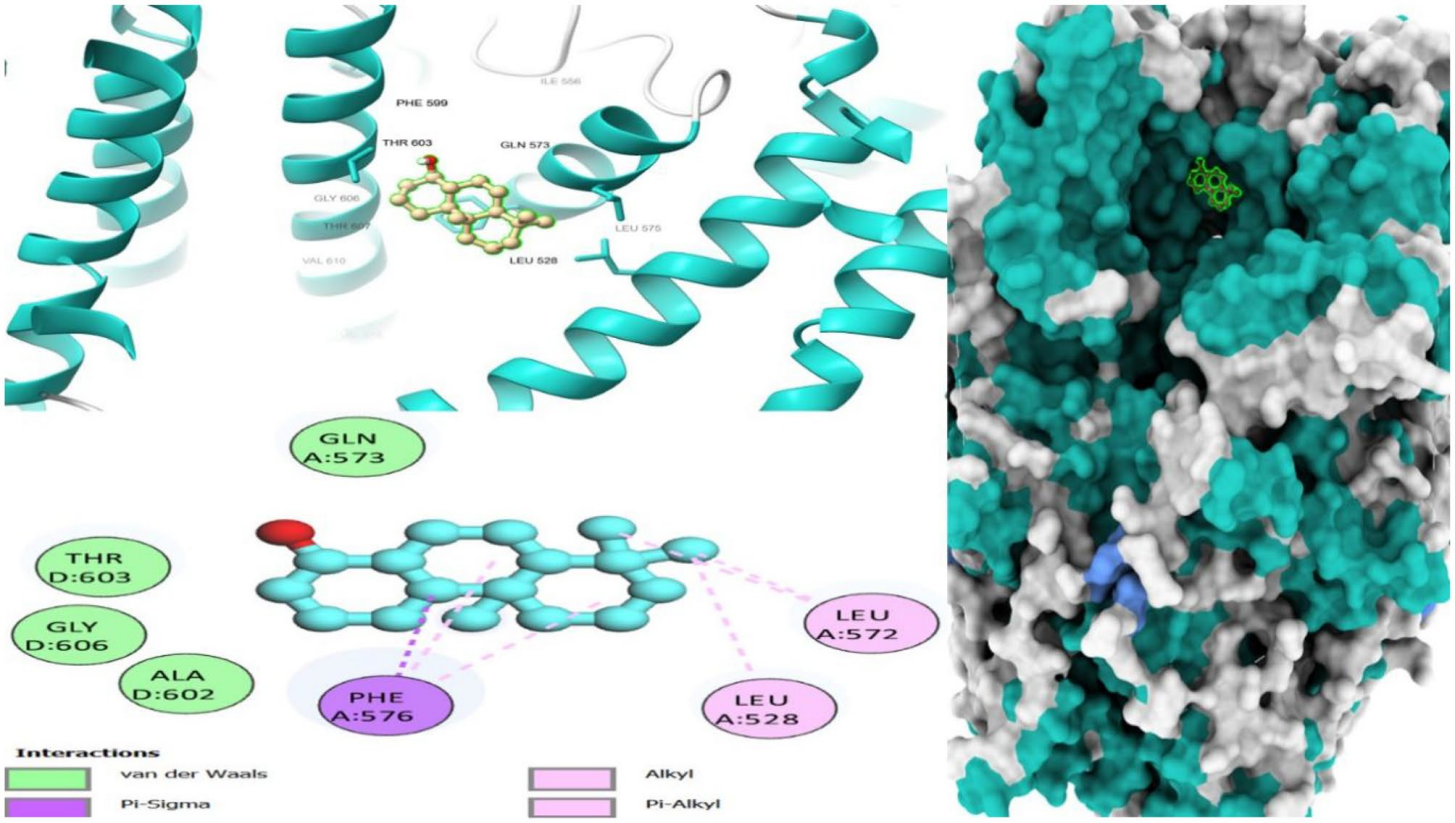
3D, 2D and mapping surface showing binding modes between 1-phenanthrenol, tetradecahydro-4b,8,8-trimethyl-, [1R-(1. alpha.,4a. beta.,4b. alpha.,8a. beta., 10a.alpha.)]-and active site residues of cold sensor protein.

**Figure 11:**
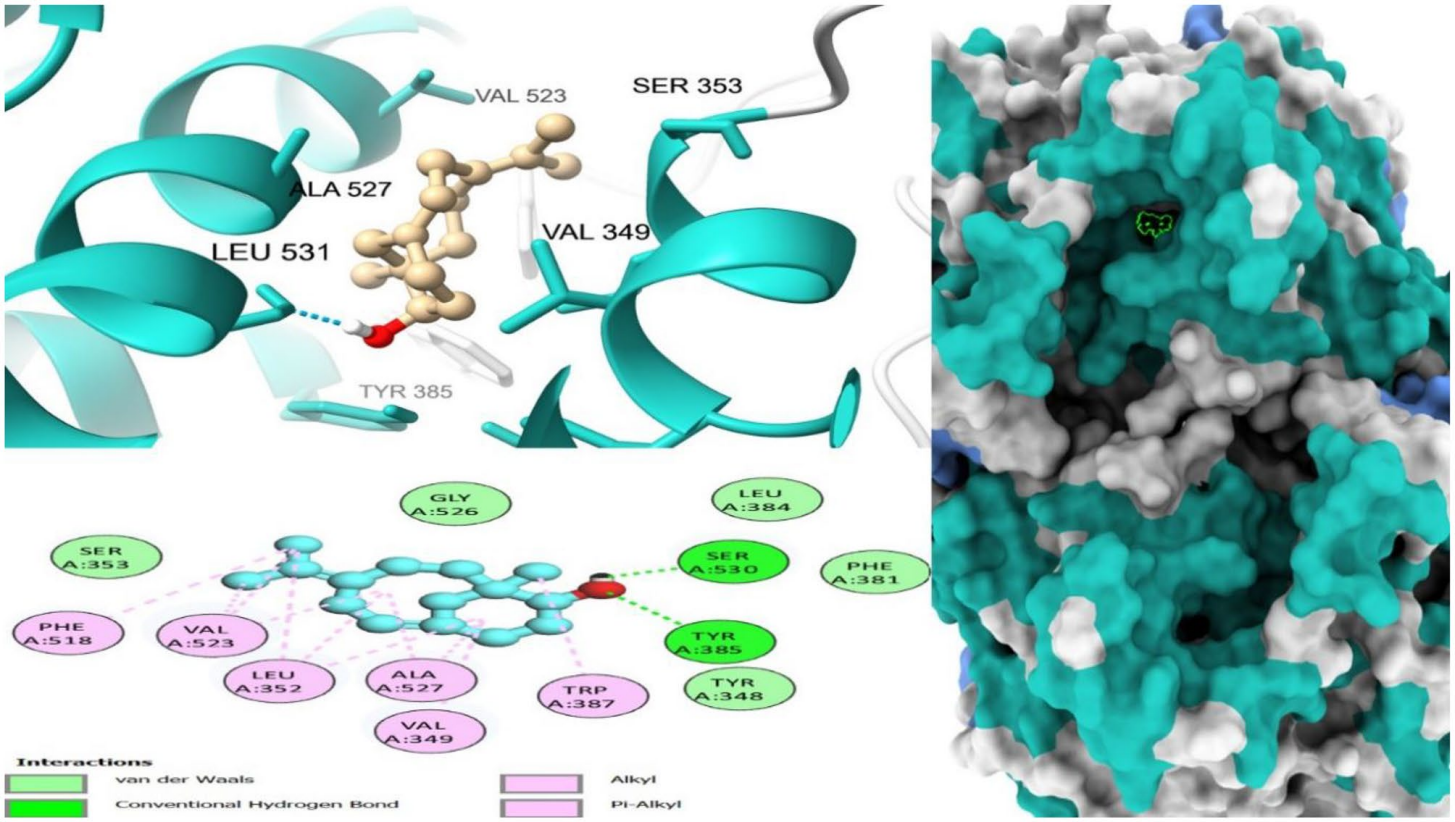
3D, 2D and mapping surface showing binding modes between 7R,8R-8-hydroxy-4-isopropylidene-7-methylbicyclo [5.3.1] undec-1-ene, and active site residues of cold sensor protein.

**Figure 12:**
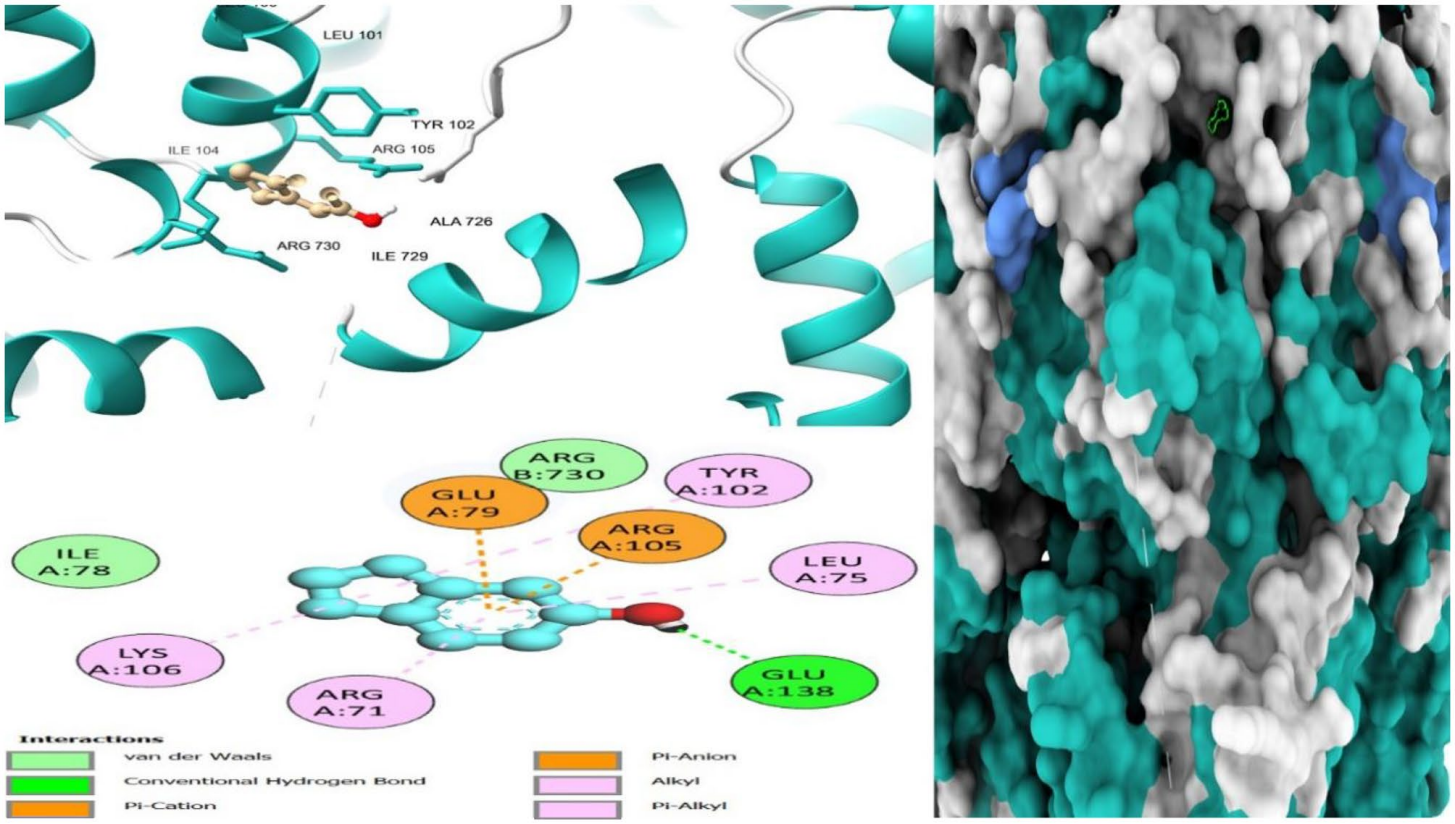
3D, 2D and mapping surface showing binding modes between 1H-inden-5-ol, 2,3-dihydro- and active site residues of cold sensor protein.

Drug-target interaction tests were also done for the molecules with the best molecular docking results to identify the functional groups, protein target residues, and functional groups involved in the interactions. The results showed that various chemical interactions were involved and these included pi-alkyl interactions, Van der Waals interactions, alkyl interactions, hydrogen bond interactions, Pi-Sigma interactions, Pi-Anion interactions, and Pi-Cation interactions. Figures 7, 8, 9, 10, 11 and 12 show the chemical interactions between the reference drug and target, as well as the compounds and target interactions and the functional groups or protein residues responsible for the chemical interactions for some of the tests that were done. Additional File: Table 8 shows all the drug-target interactions as well as the protein residues involved for the best compounds.

## Discussion

*M. crassa* (Fig. 2) is a medicinal plant used in Malawi for various medicinal purposes, but until now, there has been no reported scientific validation of its medicinal uses. Therefore, this might be the first report on the plant’s medicinal value in disease management in the dentistry or oral health fields.

Phytochemical and in silico screening were used because they are the most commonly used initial screening methods for medicinal plants that mostly contain numerous bioactive phytocompounds. This multi-composition state presents challenges when identifying bioactive compounds responsible for activity and/or reaction mechanisms. Therefore, phytochemical and in silico bioactivity screening approaches (Fig. 1) help to devise appropriate ways to discover bioactive compounds from medicinal plants. Phytochemical screening tools include GC–MS and FT-IR, and in silico screening tools for predicting pharmacokinetics and pharmacodynamic properties include SwissADME, pkCSM, and molecular docking tools such as Autodock Vina^20,32,78–80^. GC–MS has been commonly used in drug discovery from plants to identify the components in plant crude extracts, while FT-IR has been widely used to identify functional groups in both crude extracts and isolated compounds. Computational tools such as molecular docking have been used to predict compounds that can execute targeted bioactivity on drug targets of interest as well as predict the pharmacokinetic profiles. An ideal compound should have desirable pharmacological profiles such as pharmacodynamic, pharmacokinetic, and toxicity properties. This is based on various bioactivity indicators and cut-off points that are used as reference points for determining whether a compound falls within acceptable limits for druggable compounds. Therefore, the use of these tools provides baseline data for advanced drug discovery processes^35^. This study analysed the chemical composition and in silico bioactivities of *M. crassa* for potential use in the treatment of dental and oral pathologies.

Most of the functional groups identified in phytocompounds play different roles in pharmacological activities such as antimicrobial, analgesic, and anti-inflammatory activity^81^. Similarly, functional groups identified in this study (Table 1, Fig. 4) also played a significant role in the drug target interactions (Figs. 3, 7, 8, 9, 10, 11 and 12, and Additional File: Table 8). The GC-MS-identified compounds (Fig. 5a,b, Additional File: Table 1) belonged to the widely known phytochemical classes from which various drugs have been discovered such as alkaloids, flavonoids, terpenes, polyphenols, organic acids, amino acids, and others^48^. Some of the compounds were also found in the literature to possess pharmacological activities including anti-inflammatory, analgesic, and antimicrobial activity (Additional File: Table 1). Three similar reports on phytochemical, in silico, and in vitro studies of extracts and compounds (such as oleic acid and methyl stearate) identified in *M. crassa* found that the extracts containing these compounds had antioxidant and antimicrobial activities^48,70^.

Virtual screening is a technique used to discover novel ligands for protein structure and plays a significant role in structure-based drug design^48^. Based on the SwissADME, and pkCSM analyses, the studied ADME-related properties included molecular weight (MW), octanol-water partition coefficients (log P), topological polar surface area (TPSA), water solubility, gastrointestinal absorption (GIA), blood-brain barrier (BBB) and central nervous system (CNS) permeability, and glycoprotein (P-gp), and cytochrome P450s (CYPs) inhibition. Many compounds exhibited good absorption, distribution, metabolism, and excretion scores that were within the benchmarks for marketed drugs (Additional Files: Tables 3, 4, 5, and 6). For example, the log P value results were good for all compounds. This is a constant negative value for hydrophilic compounds (higher affinity for the aqueous phase), a positive value for lipophilic compounds (higher affinity for the lipid/organic solvent phase), and a zero (0) value for compounds that partition equally between lipid and aqueous phases. If log P = 1, it means the partitioning of the compound is 10 times greater in the lipid/organic solvent phase compared to the aqueous phase. In addition, Lipinski’s rule of five states that oral absorption compounds must have a log P of less than 5, while sub-lingual absorption favours a log P > 5^71,72^. Based on the way the medicinal plant is administered, which is holding a boiled mixture of the plant and water (water extract) in the mouth for a few minutes, the compounds might be absorbed in the mouth sub-lingually or through other absorption routes. Therefore, the absorption results are consistent with the way the medicinal plant is used. P-glycoprotein (P-gp) is an adenosine triphosphate (ATP)-binding cassette sub-family B member 1 (ABCB1)^82^, and it is crucial in the active transport of various substrates out of cells, resulting in poor intestinal permeation and limited oral bioavailability. Natural constituents that inhibit P-gp overcome P-gp efflux and enhance the oral absorption and bioavailability of many P-gp substrates. The co-administration of P-gp inhibitors with P-gp substrates can result in drug-herbal interactions and increased side effects due to the pharmacological activity of these substrates^83^. Knowledge of the substrate and inhibition status of the compounds is crucial in identifying potential drug-herbal interactions or therapeutic effects that can emanate from the co-administration of p-gp substrates and inhibitors. From these results, most of the compounds were neither substrates nor inhibitors, removing any drug-herbal interactions and low bioavailability fears and making the compounds identified in the extracts favorable compounds for further drug development. The fraction unbound (human) parameter predicts a fraction of a compound quantity that would be unbound in plasma. A high fraction unbound in plasma means the compound is free to leave the plasma component and travel to the site of action, while a low fraction unbound means the compound is not free to leave the plasma to reach the site of action. Predicting the fraction unbound in plasma provides a good understanding of the pharmacokinetic properties of a drug to assist candidate selection in the early stages of drug discovery^84^. Many compounds in the extracts of *M. crassa* had high fractions that were unbound and, in some cases, better than some marketed drugs, making them favourable compounds for further drug development. OCT2 in the renal excretion of cationic drugs raises the possibility of drug-herbal interactions where an inhibitor (perpetrator) drug decreases the OCT2-dependent renal clearance of a victim (substrate) drug. Elucidation of the excretion mechanisms of compounds aids in determining the possible drug-herbal interactions that would be perpetrated by the transporter inhibitors^85^. From the results, most of the compounds were not OCT2 substrates, entailing a lower chance of drug-herbal interaction through this mechanism. Bultum et al. also evaluated plant extracts that contained compounds found in *M. crassa*, palmitic acid and methyl palmitate, and the ADME results were favourable for drug development benchmarks^86^. Therefore, many compounds in the *M. crassa* extract can be taken further for drug development.

The SwissADME and pkCSM tools evaluate ADME, and the decisions made from these tools were compared for consistency (Additional File: Tables 1 and 6; Fig. 6). The results showed that the two tools had three comparable parameters. For example, the comparative analysis of log P measurements by SwissADME and pkCSM showed that they agreed on the individual level (95% limit of agreement). The agreement of the log P measurements amongst the tools used is crucial for the reliability of the results. Similar findings were observed by Dulsat et al.^87^. However, some parameters had different endpoints, which made comparison impossible, like the Chikowe et al. study that found that different in silico methods gave varying decisions as well as incomparable parameters^88^. Therefore, online tool results need to be interpreted with caution.

Molecular docking also showed that some of the compounds could be used as analgesics and anti-inflammatory agents in dentistry or oral health diseases. Notable compounds included stigmastan-3,5-diene, propane, 1,3-dichloro- and 7R,8R-8-hydroxy-4-isopropylidene-7-methylbicyclo [5.3.1] undec-1-ene (Additional File: Tables 2–6; Figs. 7, 8, 9, 10, 11 and 12). Based on GC–MS results, stigmastan-3,5-diene had the highest concentration as shown by its highest area under the peak. The study revealed that these compounds can efficiently bind to the targeted receptors associated with oral health diseases. An interesting fact about the best-performing compounds is that they can act on more than one target protein of different disease pathways related to oral health diseases (Additional File: Tables 7 and 8), which can enhance their efficacy and minimize the effects of resistance or tolerance to one target. Therefore, these bioactive compounds could be used in oral health diseases for the management of pain and inflammation, and the results support the traditional use of the plant for the same. Ralte et al. also evaluated the anti-inflammatory activity of one of the compounds found in *M. crassa* extracts, oleic acid, and found that it also had anti-inflammatory activity against the cox-2 target (− 5.3 kcal/mol), which was comparable to the results found in this study (− 6.0 kcal/mol). In this study, the highest binding affinity activity was observed in stigmastan-3,5-diene (− 9.7 kcal/mol), which was higher than isoxicam (− 9.3 kcal/mol) binding affinity, a compound that had the highest binding affinity in this similar study^48^.

Based on the review of published literature, this is the first report about the molecular docking of *M. crassa* bioactive compounds for analgesic and anti-inflammatory activity screening in oral health diseases. Therefore, the results of this study open a new frontier for targeted plants that require further studies. The literature review has also shown that the compounds in the plant have antimicrobial, antipyretic, analgesic, and anti-inflammatory activities (Additional File: Table 1), which are also relevant to oral health diseases. Therefore, the isolation of these bioactive compounds and their detailed in vivo and in vitro characterization would provide a deeper understanding of their suitability for drug discovery and development. Despite the positive results, the analyses have also shown that some compounds could be toxic, and the toxic effects include hepatotoxicity, skin sensitization, and BBB permeation (Additional File: Tables 3, 4, 5, and 6). The computational results are corroborated by the literature review results, which also showed that some of the compounds could cause toxic effects such as paralysis, primary dermal irritation, and increasing tumour growth at moderate ROS levels, as well as, neurotoxic, fetotoxic, anti-angiogenic, and cytotoxic effects (Additional File: Table 1). However, toxicity is dose-dependent, a parameter that was not studied in this research, so the toxicity results also need to be interpreted with caution. Therefore, it is important to perform toxicity tests as well in future studies and this further highlights the need for further studies on the phytocompounds in the plant.

## Conclusions

The present study identified bioactive compounds found in *M. crassa*, their respective functional groups, and their potential for biological activity with pain and inflammation protein targets, using GC-MS analysis, FT-IR analysis, and molecular docking, respectively. This was the first time this kind of study was done on compounds from *M. crassa*. Some of the identified bioactive compounds showed that they had anti-inflammatory and analgesic effects, and these included stigmastan-3,5-diene, propane, 1,3-dichloro- and 7R,8R-8-hydroxy-4-isopropylidene-7-methylbicyclo [5.3.1] undec-1-ene. The literature and computational analyses have also shown that some of the phytocompounds in the plant could be toxic. However, the results need to be interpreted with caution because some of the factors that affect phytocompound efficacy and toxicity, such as dose were not evaluated in the study. Overall, the results support the traditional use of the plant for oral pain and inflammation. The results also support the need for in vitro and in vivo studies and further computational studies to validate the study results and elucidate the mechanism of action of the bioactive phytocompounds.

### Supplementary Information

Supplementary Table 1.

Supplementary Table 2.

Supplementary Tables.

Supplementary Table 6.

Supplementary Table 7.

Supplementary Table 8.

## Data Availability

All data generated or analysed during this study are included in this published article and its supplementary information files.

## Abbreviations

ADMET: Absorption, distribution, metabolism, excretion, and toxicity
DCM: Dichloromethane
DLATGS/DLTGS: Deuterated l-alanine doped triglycine sulphate
ESOL: Estimating aqueous solubility
FRIM: Forest Research Institute of Malawi
FT-IR: Fourier transform infrared
GC–MS: Gas chromatography-mass spectrometry
HBA: Hydrogen bond acceptor
HBD: Hydrogen bond donor
HIA: Human intestinal absorption
KBr: Potassium bromide
LMICs: Low to middle-income countries
MeOH: Methanol
MSD: Mass spectrometer detector
NHBG: National Herbarium and Botanical Gardens
NIST: National Institute of Standards and Technology
RMSD: Root mean square deviation
TPSA: Topological polar surface area
WHO: World Health Organization
