# Supplementary material for: GC–MS analysis, molecular docking, and pharmacokinetic studies of Multidentia crassa extracts’ compounds for analgesic and anti-inflammatory activities in dentistry
Source: Sci Rep. 2024 Jan 22;14:1876. doi: 10.1038/s41598-023-47737-x (PMC10803350; doi:10.1038/s41598-023-47737-x)
Supplement: Supplementary file 1 — Supplementary Table 1. [file 41598_2023_47737_MOESM1_ESM.docx]

**Additional File: Table 1_GCMS results for the Dichloromethane and Methanol extracts of** *Multidentia crassa* (Hiern) Bridson & Verdc

| **S.No.** | **RT** | **Compound Name** | **IUPAC Name** | **Mol.Weight** | **Mol.Formula** | **Functional Groups** | **Bioactivity** | **Toxicity** |
| --- | --- | --- | --- | --- | --- | --- | --- | --- |
| DICHLOROMETHANE | | | | | | | | |
| 1 | 5.196 | 1,3-Dichloropropane | 1,3-dichloropropane | 112.98 | C_3_H_6_Cl_2_ | Alkyl halide, chlorine, alkane | No information | No information |
| 2 | 8.261 | 5-Indanol | 2,3-dihydro-1H-inden-5-ol | 134.17 | C_9_H_10_O | Benzene, Aldehyde | No information | No information |
| 3 | 8.261 | Terephthalaldehyde | terephthalaldehyde | 134.13 | C_8_H_6_O_2_ | Cycloalkane, benzene, phenol, alkanol | No information | No information |
| 4 | 8.261 | Isophthalaldehyde | benzene-1,3-dicarbaldehyde | 134.13 | C_8_H_6_O_2_ | Benzene, aldehyde | No information | No information |
| 5 | 8.903 | 1,3-Di-tert-butylbenzene | 1,3-ditert-butylbenzene | 190.32 | C_14_H_22_ | Benzene | No information | No information |
| 6 | 8.903 | Precocene I | 7-methoxy-2,2-dimethylchromene | 190.24 | C_12_H_14_O_2_ |  |  |  |
| 7 | 8.903 | 1,4-Di-tert-butylbenzene | 1,4-ditert-butylbenzene | 190.32 | C_14_H_22_ | Benzene, cycloalkane, ether, , clyclic ether | Antibacterial activity (Kouame et al., 2018) | No information |
| 8 | 9.545 | Tridecane | tridecane | 184.36 | C_13_H_28_ | Alkane | Antibacterial, antioxidant, and antiproliferative against HeLa cells (Pucot et al., 2021) | No information |
| 9 | 10.747 | beta-Elemene | (1S,2S,4R)-1-ethenyl-1-methyl-2,4-bis(prop-1-en-2-yl)cyclohexane | 204.35 | C_15_H_24_ | Cyclalkene, alkene | No information | No information |
| 10 | 10.747 | 8-Isopropenyl-1,5-dimethyl-cyclodeca-1,5-diene | (1E,5Z)-1,5-dimethyl-8-prop-1-en-2-ylcyclodeca-1,5-diene | 204.35 | C_15_H_24_ | Alkene, cyclic alkene | No information | No information |
| 11 | 10.747 | (+)-Helminthogermacrene | (1E,5Z,8S)-1,5-dimethyl-8-prop-1-en-2-ylcyclodeca-1,5-diene | 204.35 | C_15_H_24_ | Cyloalkane, alkene | No information | No information |
| 12 | 10.85 | Tetradecane | tetradecane | 198.39 | C_14_H_30_ | Alkane | Antifungal, antibacterial (Nimbeshaho et al., 2020) | No information |
| 13 | 11.264 | 2-Isopropenyl-1-methyl-4-(1-methylethylidene)-1-vinylcyclohexane | (1S,2S)-1-ethenyl-1-methyl-4-propan-2-ylidene-2-prop-1-en-2-ylcyclohexane | 204.35 | C_15_H_24_ | cycloalkane, alkene | Anticancer activity (Zhai et al., 2019) | Fetotoxic and Anti-angiogenic (Dosoky and Setzer, 2021) |
| 14 | 11.492 | Humulene | (1E,4E,8E)-2,6,6,9-tetramethylcycloundeca-1,4,8-triene | 204.35 | C_15_H_24_ | cycloalkene | topical and systemic anti-inflammatory, analgesic topically, orally, or by aerosol, antineoplastic effect (Richter et al., 2021) | Increases tumor growth at moderate ROS levels |
| 15 | 11.658 | alpha-Longipinene | 2,6,6,9-tetramethyltricyclo[5.4.0.02,8]undec-9-ene | 204.35 | C_15_H_24_ | Cycloalkane, cycloalkene | No information | No information |
| 16 | 11.658 | Cedrene | 2,6,6,8-tetramethyltricyclo[5.3.1.01,5]undec-8-ene | 204.35 | C_15_H_24_ | Cycloalkane, cycloalkane | No information | No information |
| 17 | 11.782 | gamma-Muurolene | (1R,4aR,8aS)-7-methyl-4-methylidene-1-propan-2-yl-2,3,4a,5,6,8a-hexahydro-1H-naphthalene | 204.35 | C_15_H_24_ | cycloalkane, cycloalkane, alkane, alkene | No information | No information |
| 18 | 11.782 | 1,2,4a,5,6,8a-Hexahydro-1-isopropyl-4,7-dimethylnaphthalene | 4,7-dimethyl-1-propan-2-yl-1,2,4a,5,6,8a-hexahydronaphthalene | 204.35 | C_15_H_24_ | Cycloalkane, alkane | No information | No information |
| 19 | 11.886 | (-)-Alloaromadendrene | (1aR,4aS,7R,7aR,7bS)-1,1,7-trimethyl-4-methylidene-2,3,4a,5,6,7,7a,7b-octahydro-1aH-cyclopropa[e]azulene | 204.35 | C_15_H_24_ | cycloalkene, alkene, alkane | Topical and systemic nti-inflammatory activity (=dexamethasone in an animal model , analgesic (topically, orally, or by aerosol, antineoplastic, wound healing (Richter et al., 2021) | Paralysis and muscle contractions (insects) or neurotoxic effects (Agus, 2021) |
| 20 | 11.886 | beta-Humulene | (1E,5E)-1,4,4-trimethyl-8-methylidenecycloundeca-1,5-diene | 204.35 | C_15_H_24_ | Alkene, cyclo alkane | No information | No information |
| 21 | 11.989 | 2-Isopropenyl-4a,8-dimethyl-1,2,3,4,4a,5,6,7-octahydronaphthalene | 4a,8-dimethyl-2-prop-1-en-2-yl-2,3,4,5,6,7-hexahydro-1H-naphthalene | 204.35 | C_15_H_24_ | Cycloalkane, cycloalkene | No information | no information |
| 22 | 11.989 | Selina-4(15),7(11)-diene | (4aS,8aR)-8a-methyl-4-methylidene-6-propan-2-ylidene-2,3,4a,5,7,8-hexahydro-1H-naphthalene | 204.35 | C_15_H_24_ | Cycloalkane, alkene | No information | No information |
| 23 | 12.238 | 2,5-Di-tert-butylphenol | 2,5-ditert-butylphenol | 206.32 | C_14_H_22_O | Benzene, phenol, alkane, alkanol | anti-bacterial activity, antifungal activity and antioxidant, anti-inflammatory activity, anticancerous (Mohiuddin et al., 2018) | No information |
| 24 | 12.238 | 3,5-Di-tert-butylphenol | 3,5-ditert-butylphenol | 206.32 | C_14_H_22_O | Alkane, benzene, phenol, alkanol | Antioxidants, Antimicrobial (Addai et al., 2022) | No information |
| 25 | 12.362 | (+)-delta-Cadinene | (1S,8aR)-4,7-dimethyl-1-propan-2-yl-1,2,3,5,6,8a-hexahydronaphthalene | 204.35 | C_15_H_24_ | Cycloalkane, alkane | no information | no information |
| 26 | 12.362 | (+)-alpha-Muurolene | (1R,4aR,8aS)-4,7-dimethyl-1-propan-2-yl-1,2,4a,5,6,8a-hexahydronaphthalene | 204.35 | C_15_H_24_ | Cycloalkane, alkane | no information | no information |
| 27 | 12.486 | 4a,5-Dimethyl-3-(prop-1-en-2-yl)-1,2,3,4,4a,5,6,7-octahydronaphthalene | 4a,5-dimethyl-3-prop-1-en-2-yl-2,3,4,5,6,7-hexahydro-1H-naphthalene | 204.35 | C_15_H_24_ | Cycloalkane | No information | No information |
| 28 | 12.486 | (+)-Aromadendrene | (1aR,4aR,7R,7aR,7bS)-1,1,7-trimethyl-4-methylidene-2,3,4a,5,6,7,7a,7b-octahydro-1aH-cyclopropa[e]azulene | 204.35 | C_15_H_24_ | Alkene, cycloalkane | No information | No information |
| 29 | 12.486 | (+)-Cyclosativene | 1,2-dimethyl-8-propan-2-yltetracyclo[4.4.0.02,4.03,7]decane | 204.35 | C_15_H_24_ | Cycloalkane, cycloalkane, alkene | No information | No information |
| 30 | 12.569 | 3,7(11)-Eudesmadiene | 5,8a-dimethyl-3-propan-2-ylidene-1,2,4,4a,7,8-hexahydronaphthalene | 204.35 | C_15_H_24_ | Cycloalkane, alkane | No information | No information |
| 31 | 12.569 | Epizonarene | 1,6-dimethyl-4-propan-2-yl-1,2,3,7,8,8a-hexahydronaphthalene | 204.35 | C_15_H_24_ | Cycloalkane, cycloalkane, alkene | No information | No information |
| 32 | 12.755 | (-)-alpha-Gurjunene | (1aR,4R,7bS)-1,1,4,7-tetramethyl-1a,2,3,4,4a,5,6,7b-octahydrocyclopropa[e]azulene | 204.35 | C_15_H_24_ | Cycloalkane | No information | No information |
| 33 | 12.755 | Patchoulene | (1R,5R,8S)-1,5,11,11-tetramethyltricyclo[6.2.1.02,6]undec-2(6)-ene | 204.35 | C_15_H_24_ | Alkane | inflammatory activity, down-regulation of protein expression of iNOS and COX-2 (Liang et al., 2017) | No information |
| 34 | 13.211 | 1H-Benzocyclohepten-7-ol, 2,3,4,4a,5,6,7,8-octahydro-1,1,4a,7-tetramethyl-, cis- | (7S)-4,4,7,9a-tetramethyl-1,2,3,6,8,9-hexahydrobenzo[7]annulen-7-ol | 222.37 | C_15_H_26_O | Cycloalkene, Alkanol, cycloalkane | No information | No information |
| 35 | 13.315 | Patchoulane | 4,10,11,11-tetramethyltricyclo[5.3.1.01,5]undecane | 206.37 | C_15_H_26_ | Cycloalkane | No information | No information |
| 36 | 13.315 | 3,4-Dimethyl-3-cyclohexenylmethanal | 3,4-dimethylcyclohex-3-ene-1-carbaldehyde | 138.21 | C_9_H_14_O | Aldehyde, cyclic alkene | No information | No information |
| 37 | 13.687 | (-)-gamma-Cadinene | (1R,4aS,8aS)-7-methyl-4-methylidene-1-propan-2-yl-2,3,4a,5,6,8a-hexahydro-1H-naphthalene | 204.35 | C_15_H_24_ | Cycloalkane, alkene, cycloalkene | No information | No information |
| 38 | 13.832 | alpha-Cadinene | (1S,4aR,8aR)-4,7-dimethyl-1-propan-2-yl-1,2,4a,5,6,8a-hexahydronaphthalene | 204.35 | C_15_H_24_ | Cycloalkane | No information | No information |
| 39 | 15.034 | 7R,8R-8-Hydroxy-4-isopropylidene-7-methylbicyclo[5.3.1]undec-1-ene | (1Z,7R,8R)-7-methyl-4-propan-2-ylidenebicyclo[5.3.1]undec-1-en-8-ol | 220.35 | C_15_H_24_O | Alkanol, cycloalkane, cycloalkane, alkene | Antioxidant (Sankpal, 2022) | No information |
| 40 | 15.406 | Octadecane | octadecane | 254.50 | C_18_H_38_ | Alkane | Antimicrobial activity (Rouis-Soussi et al., 2014) | No information |
| 41 | 16.98 | (1E,5E,11E)-1,5,11-Trimethyl-8-isopropenylcyclotetradeca-1,5,11-triene | (1Z,5Z,9Z)-1,5,9-trimethyl-12-prop-1-en-2-ylcyclotetradeca-1,5,9-triene | 272.50 | C_20_H_32_ | Cycloalkalkene, alkene | No information | No information |
| 42 | 17.374 | Eicosane | icosane | 282.50 | C_20_H_42_ | alkane | Antifungal (Ahsan et al., 2017),anti-inflammatory, analgesic, and antipyretic effects (Okechukwu, 2020) | No toxicity to rats at a concentration of 1000 mg/kg (Okechukwu, 2020) |
| 43 | 21.971 | Bis(2-ethylhexyl) phthalate | bis(2-ethylhexyl) benzene-1,2-dicarboxylate | 390.60 | C_24_H_38_O_4_ | Benzene, ester, alkane | Antishigellosis activity, proinflammatory activity, antibacterial activity (Habib and Karim, 2009), antimutagenic(Cruz-Ramirez et al., 2021), cytotoxic (Momen et al., 2018) | Cytotoxic |
| 44 | 21.971 | Diisooctyl phthalate | bis(6-methylheptyl) benzene-1,2-dicarboxylate | 390.60 | C_24_H_38_O_4_ | Benzene, ester, alkene | No information | Primary dermal irritant (Versar Inc) |
| 45 | 21.971 | Phthalic acid, di(2-propylphenyl) ester | bis(2-propylphenyl) benzene-1,2-dicarboxylate | 402.50 | C_26_H_26_O_4_ | Benzene, ester, alkane | antimicrobial activities (Osuntokun and Cristina, 2019) | No information |
| 46 | 26.776 | 2-Amino-3,5-dibromopyridine | 3,5-dibromopyridin-2-amine | 251.91 | C_5_H_4_Br_2_N_2_ | Pyridine, amine, bromine | No information | No information |
| METHANOL EXTRACT | | | | | | | | |
| 47 | 4.617 | Succinic acid, hex-4-yn-3-yl pentyl ester | 4-O-hex-4-yn-3-yl 1-O-pentyl butanedioate | 268.35 | C_18_H_34_O_2_ | Alkane, ester, alkyne | No information | No information |
| 48 | 16.67 | Methyl palmitate | methyl hexadecanoate | 270.5 | C_16_H_32_O_2_ | ester, alkane | Anti-inflammatory, hypocholesterolemic, cancer preventive, hepatoprotective, nematicide, insectifuge, antihistaminic, antieczemic, antiacne, alpha reductase inhibitor, antiandrogenic, antiarthritic, anticoronary (Krishnamoorthy and Subramaniam, 2014) | No information |
| 49 | 17.125 | Palmitic Acid | hexadecanoic acid | 256.42 | C_17_H_34_O_2_ | carboxylic acid, alkene | Anti-inflammatory, Antiandrogenic, Cancer preventive, Dermatitigenic, Hypocholesterolemic, 5-Alpha reductase inhibitor, Anemiagenic, Insectifuge, Flavor(Natarajan et al.,, 2019) | No information |
| 50 | 18.285 | 11-Octadecenoic acid methyl ester | methyl octadec-11-enoate | 296.5 | C_18_H_34_O_2_ | Ester carboxylic acid | Antioxidant and antimicrobial activities (Rahman et al., 2014) | No information |
| 51 | 18.285 | Methyl elaidate | methyl (E)-octadec-9-enoate | 296.5 | C_19_H_38_O_2_ | carboxylic acid, alkane | antioxidant, anti-inflammatory, hypocholesterolemic and cancer prevention activities (Mazumder et al., 2020) | No information |
| 52 | 18.285 | Methyl oleate | methyl (Z)-octadec-9-enoate | 296.5 | C_19_H_36_O_2_ | Ester, alkene | Antioxidant activity (Rahman et al., 2014) | No information |
| 53 | 18.533 | Methyl Stearate | methyl octadecanoate | 298.5 | C_29_H_48_ | Ester, alkene | Anti-inflammatory, Antiandrogenic Cancer preventive, DermatitigenicHypocholesterolemic,5-Alphareductase inhibitor, Anemiagenic Insectifuge, Flavor (Natarajan et al., 2019) | No information |
| 54 | 18.72 | 9-Octadecenoic acid | octadec-9-enoic acid | 282.5 | C_19_H_36_O_2_ | alkane, alkene, carboxylic acid | Antibacterial (Dilika et al., 2000) | No information |
| 55 | 18.72 | Oleic Acid | (Z)-octadec-9-enoic acid | 282.5 | C_18_H_34_O_2_ | ester, alkane | Antimicrobial activity(Nakaziba et al., ), antioxidant (abdel hady et al., 2018) | No information |
| 56 | 18.72 | Elaidic Acid | (E)-octadec-9-enoic acid | 282.5 | C_19_H_36_O_2_ | Carboxylic acid, alkane, alkene | Anti-inflammatory, Antiandrogenic, Cancer preventive, Dermatitigenic, Hypocholesterolemic, 5-Alpha reductase inhibitor, Anemiagenic Insectifuge, Flavor (Natarajan et al., 2019) | No information |
| 57 | 22.199 | Stigmastan-3,5-diene | 17-(5-ethyl-6-methylheptan-2-yl)-10,13-dimethyl-2,7,8,9,11,12,14,15,16,17-decahydro-1H-cyclopenta[a]phenanthrene | 396.7 | C_17_H_30_O | cycloalkane, cycloalkane, alkane, steroid | Antimicrobial (Diab et al., 2021) | No information |
| 58 | 22.882 | Podocarpan-14beta-ol | (1R,4aS,4bR,8aS,10aR)-4b,8,8-trimethyl-1,2,3,4,4a,5,6,7,8a,9,10,10a-dodecahydrophenanthren-1-ol | 250.4 | C_15_H_24_O_4_ | Cyclic alkane, cyclic alkene, carboxylix acid, alkanol | No information | No information |
