# Supplementary material for: GC–MS analysis, molecular docking, and pharmacokinetic studies of Multidentia crassa extracts’ compounds for analgesic and anti-inflammatory activities in dentistry
Source: Sci Rep. 2024 Jan 22;14:1876. doi: 10.1038/s41598-023-47737-x (PMC10803350; doi:10.1038/s41598-023-47737-x)
Supplement: Supplementary file 2 — Supplementary Table 2. [file 41598_2023_47737_MOESM2_ESM.docx]

**Additional File: Table 2_pkCSM and SwissADME-derived Molecular properties of the compounds identified by the GC-MS analysis.**

| **Extract** | **S.No.** | **Compound Name** | **MOL_WEIGHT** | **LOGP** | **#ROTATABLE_BONDS** | **HBAs** | **HBDs** | **SURFACE_AREA (pkCSM/SwissADME)** | **# OF LIPINSKI FAILS** | **LIPINSKI EVALUATION** | **# OF VEBER FAILS** | **VEBER EVALUATION** | **Overall** |
| --- | --- | --- | --- | --- | --- | --- | --- | --- | --- | --- | --- | --- | --- |
| DICHLOROMETHANE EXTRACT | 1 | 5-Indanol | 134.17 | 1.8809 | 0 | 1 | 1 | 60.315/20.23 | 0 | Pass | 0 | Pass | Pass |
|  | 2 | 1,4-Di-tert-butylbenzene | 190.33 | 4.2816 | 0 | 0 | 0 | 88.351 | 0 | Pass | 0 | Pass | Pass |
|  | 3 | 2-Isopropenyl-1-methyl-4-(1-methylethylidene)-1-vinylcyclohexane | 204.357 | 4.8913 | 2 | 0 | 0 | 94.774 | 0 | Pass | 0 | Pass | Pass |
|  | 4 | 8-Isopropenyl-1,5-dimethyl-cyclodeca-1,5-diene | 204.357 | 5.0354 | 1 | 0 | 0 | 94.774 | 1 | Pass | 0 | Pass | Pass |
|  | 5 | 2,5-Di-tert-butylphenol | 206.329 | 3.9872 | 0 | 1 | 1 | 93.145 | 0 | Pass | 0 | Pass | Pass |
|  | 6 | Precocene I | 190.242 | 2.8794 | 1 | 2 | 0 | 84.153/18.46 | 0 | Pass | 0 | Pass | Pass |
|  | 7 | beta-Elemene | 204.357 | 4.7472 | 3 | 0 | 0 | 94.774 | 0 | Pass | 0 | Pass | Pass |
|  | 8 | Tridecane | 184.367 | 5.3173 | 10 | 0 | 0 | 85.119 | 1 | Pass | 1 | Fail | Fail |
|  | 9 | 1,2,4a,5,6,8a-Hexahydro-1-isopropyl-4,7-dimethylnaphthalene | 204.357 | 4.5811 | 1 | 0 | 0 | 94.458 | 0 | Pass | 0 | Pass | Pass |
|  | 10 | 1,3-Di-tert-butylbenzene | 190.33 | 4.2816 | 0 | 0 | 0 | 88.351 | 0 | Pass | 0 | Pass | Pass |
|  | 11 | Humulene | 204.357 | 5.0354 | 0 | 0 | 0 | 94.774 | 1 | Pass | 0 | Pass | Pass |
|  | 12 | Cedrene | 204.357 | 4.415 | 0 | 0 | 0 | 94.141 | 0 | Pass | 0 | Pass | Pass |
|  | 13 | gamma-Muurolene | 204.357 | 4.5811 | 1 | 0 | 0 | 94.458 | 0 | Pass | 0 | Pass | Pass |
|  | 14 | (-)-Alloaromadendrene | 204.357 | 4.2709 | 0 | 0 | 0 | 94.141 | 0 | Pass | 0 | Pass | Pass |
|  | 15 | beta-Humulene | 204.357 | 5.0354 | 0 | 0 | 0 | 94.774 | 0 | Pass | 0 | Pass | Pass |
|  | 16 | 2-Isopropenyl-4a,8-dimethyl-1,2,3,4,4a,5,6,7-octahydronaphthalene | 204.357 | 4.8693 | 1 | 0 | 0 | 94.458 | 0 | Pass | 0 | Pass | Pass |
|  | 17 | Selina-4(15),7(11)-diene | 204.35 7 | 4.8693 | 0 | 0 | 0 | 94.458 | 0 | Pass | 0 | Pass | Pass |
|  | 18 | 3,5-Di-tert-butylphenol | 206.329 | 3.9872 | 0 | 1 | 1 | 93.145/20.23 | 0 | Pass | 0 | Pass | Pass |
|  | 19 | (+)-delta-Cadinene | 204.357 | 4.7252 | 1 | 0 | 0 | 94.458 | 0 | Pass | 0 | Pass | Pass |
|  | 20 | (+)-alpha-Muurolene | 204.357 | 4.5811 | 1 | 0 | 0 | 94.458 | 0 | Pass | 0 | Pass | Pass |
|  | 21 | 4a,5-Dimethyl-3-(prop-1-en-2-yl)-1,2,3,4,4a,5,6,7-octahydronaphthalene | 204.357 | 4.7252 | 1 | 0 | 0 | 94.458 | 0 | Pass | 0 | Pass | Pass |
|  | 22 | (+)-Aromadendrene | 204.35 7 | 4.2709 | 0 | 0 | 0 | 94.141 | 0 | Pass | 0 | Pass | Pass |
|  | 23 | 3,7(11)-Eudesmadiene | 204.35 7 | 4.8693 | 0 | 0 | 0 | 94.458 | 0 | Pass | 0 | Pass | Pass |
|  | 24 | (-)-alpha-Gurjunene | 204.357 | 4.415 | 0 | 0 | 0 | 94.141 | 0 | Pass | 0 | Pass | Pass |
|  | 25 | Patchoulene | 204.357 | 4.5591 | 0 | 0 | 0 | 94.141 | 0 | Pass | 0 | Pass | Pass |
|  | 26 | 1H-Benzocyclohepten-7-ol, 2,3,4,4a,5,6,7,8-octahydro-1,1,4a,7-tetramethyl-, cis- | 222.372 | 4.0641 | 0 | 1 | 1 | 99.941/20.23 | 0 | Pass | 0 | Pass | Pass |
|  | 27 | Patchoulane | 206.373 | 4.4949 | 0 | 0 | 0 | 94.831 | 0 | Pass | 0 | Pass | Pass |
|  | 28 | 3,4-Dimethyl-3-cyclohexenylmethanal | 138.21 | 2.3218 | 1 | 1 | 0 | 62.125/17.07 | 0 | Pass | 0 | Pass | Pass |
|  | 29 | (-)-gamma-Cadinene | 204.357 | 4.5811 | 1 | 0 | 0 | 94.458 | 0 | Pass | 0 | Pass | Pass |
|  | 30 | alpha-Cadinene | 204.357 | 4.5811 | 1 | 0 | 0 | 94.458 | 0 | Pass | 0 | Pass | Pass |
|  | 31 | 7R,8R-8-Hydroxy-4-isopropylidene-7-methylbicyclo[5.3.1]undec-1-ene | 220.356 | 3.9842 | 0 | 1 | 1 | 99.252/20.23 | 0 | Pass | 0 | Pass | Pass |
|  | 32 | Octadecane | 254.502 | 7.2678 | 15 | 0 | 0 | 116.943 | 1 | Pass | 0 | Pass | Pass |
|  | 33 | (1E,5E,11E)-1,5,11-Trimethyl-8-isopropenylcyclotetradeca-1,5,11-triene | 272.476 | 6.7619 | 1 | 0 | 0 | 125.909 | 1 | Pass | 0 | Pass | Pass |
|  | 34 | Diisooctyl phthalate | 390.564 | 6.433 | 14 | 4 | 0 | 170.550/52.6 | 1 | Pass | 2 | Fail | Fail |
|  | 35 | Phthalic acid, di(2-propylphenyl) ester | 402.49 | 6.03 | 8 | 4 | 0 | 177.015 | 1 | Pass | 1 | Fail | Fail |
|  | 36 | 2-Amino-3,5-dibromopyridine | 251.909 | 2.1888 | 0 | 2 | 1 | 69.726/38.91 | 0 | Pass | 0 | Pass | Pass |
|  | 37 | (+)-Helminthogermacrene | 204.357 | 5.0354 | 1 | 0 | 0 | 94.774 | 1 | Pass | 0 | Pass | Pass |
|  | 38 | Eicosane | 282.556 | 8.048 | 17 | 0 | 0 | 129.673 | 1 | Pass | 1 | Fail | Fail |
|  | 39 | (+)-Cyclosativene | 204.357 | 3.9607 | 1 | 0 | 0 | 93.825 | 0 | Pass | 0 | Pass | Pass |
|  | 40 | alpha-Longipinene | 204.357 | 4.415 | 0 | 0 | 0 | 94.141 | 0 | Pass | 0 | Pass | Pass |
|  | 41 | Isophthalaldehyde | 134.134 | 1.3116 | 2 | 2 | 0 | 58.484/34.1 | 0 | Pass | 0 | Pass | Pass |
|  | 42 | Epizonarene | 204.357 | 4.7252 | 1 | 0 | 0 | 94.458 | 0 | Pass | 0 | Pass | Pass |
|  | 43 | 1,3-Dichloropropane | 112.987 | 1.8541 | 2 | 0 | 0 | 42.076 | 0 | Pass | 0 | Pass | Pass |
|  | 44 | Terephthalaldehyde | 134.134 | 1.3116 | 2 | 2 | 0 | 58.484/34.1 | 0 | Pass | 0 | Pass | Pass |
|  | 45 | Tetradecane | 198.394 | 5.7074 | 11 | 0 | 0 | 91.483 | 1 | Pass | 0 | Pass | Pass |
|  | 46 | Bis(2-ethylhexyl) phthalate | 390.564 | 6.433 | 14 | 4 | 0 | 170.550/52.6 | 1 | Pass | 2 | Fail | Fail |
| METHANOL EXTRACT | 47 | Podocarpan-14beta-ol | 250.426 | 4.39 | 0 | 1 | 1 | 112.355/20.23 | 0 | Pass | 0 | Pass | Pass |
|  | 48 | Palmitic Acid | 256.43 | 5.5523 | 14 | 1 | 1 | 113.169/37.3 | 1 | Pass | 1 | Fail | Fail |
|  | 49 | Succinic acid, hex-4-yn-3-yl pentyl ester | 268.353 | 2.8451 | 9 | 4 | 0 | 115.398/52.6 | 0 | Pass | 0 | Pass | Pass |
|  | 50 | Methyl palmitate | 270.457 | 5.6407 | 14 | 2 | 0 | 119.853/26.3 | 1 | Pass | 1 | Fail | Fail |
|  | 51 | Oleic Acid | 282.468 | 6.1085 | 15 | 1 | 1 | 125.209/37.3 | 1 | Pass | 1 | Fail | Fail |
|  | 52 | 9-Octadecenoic acid | 282.468 | 6.1085 | 15 | 1 | 1 | 125.209 | 1 | Pass | 1 | Fail | Fail |
|  | 53 | Elaidic Acid | 282.468 | 6.1085 | 15 | 1 | 1 | 125.209/37.5 | 1 | Pass | 1 | Fail | Fail |
|  | 54 | Methyl elaidate | 296.495 | 6.1969 | 15 | 2 | 0 | 131.894 | 1 | Pass | 1 | Fail | Fail |
|  | 55 | Methyl oleate | 296.495 | 6.1969 | 15 | 2 | 0 | 131.894/26.3 | 1 | Pass | 1 | Fail | Fail |
|  | 56 | 11-Octadecenoic acid methyl ester | 296.495 | 6.1969 | 15 | 2 | 0 | 131.894/26.3 | 1 | Pass | 1 | Fail | Fail |
|  | 57 | Methyl Stearate | 298.511 | 6.4209 | 16 | 2 | 0 | 132.583/26.3 | 1 | Pass | 1 | Fail | Fail |
|  | 58 | Stigmastan-3,5-diene | 396.703 | 8.83 | 6 | 0 | 0 | 181.555 | 1 | Pass | 1 | Fail | Fail |
