# Supplementary material for: GC–MS analysis, molecular docking, and pharmacokinetic studies of Multidentia crassa extracts’ compounds for analgesic and anti-inflammatory activities in dentistry
Source: Sci Rep. 2024 Jan 22;14:1876. doi: 10.1038/s41598-023-47737-x (PMC10803350; doi:10.1038/s41598-023-47737-x)
Supplement: Supplementary file 3 — Supplementary Tables. [file 41598_2023_47737_MOESM3_ESM.docx]

**Additional File: Table 3a_Absorption and Distribution properties of the identified compounds using pkCSM tool**

| Extract | S.No. | Compound Name | Absorption | | | | | | | Distribution | | | |
| --- | --- | --- | --- | --- | --- | --- | --- | --- | --- | --- | --- | --- | --- |
|  |  |  | Water  solubility | Caco2  permeability | Intestinal  absorption (human) | Skin  Permeability | P-glycoprotein  substrate | P-glycoprotein I  inhibitor | P-glycoprotein II  inhibitor | VDss (human) | Fraction unbound  (human) | BBB  permeability | CNS  permeability |
|  |  |  | (log mol/L) | (log Papp  in 10-6 cm/s) | (% Absorbed) | (log Kp) | (Yes/No) | (Yes/No) | (Yes/No) | (log L/kg) | (Fu) | (log BB) | (log PS) |
| Dichloromethane Extract | 1 | 1,3-Dichloropropane | -1.943 | 1.412 | 94.969 | -1.979 | Yes | No | No | 0.045 | 0.611 | 0.626 | -2.064 |
|  | 2 | 5-Indanol | -1.728 | 1.433 | 93.01 | -1.743 | No | No | No | 0.542 | 0.382 | 0.459 | -1.6 |
|  | 3 | Terephthalaldehyde | -0.77 | 1.763 | 87.898 | -2.112 | No | No | No | -0.146 | 0.447 | -0.163 | -1.943 |
|  | 4 | Isophthalaldehyde | -0.778 | 1.763 | 87.684 | -2.137 | No | No | No | -0.129 | 0.447 | -0.163 | -1.928 |
|  | 5 | 1,3-Di-tert-butylbenzene | -5.965 | 1.503 | 94.226 | -1.86 | No | No | No | 0.954 | 0 | 0.569 | -0.785 |
|  | 6 | Precocene I | -3.109 | 1.262 | 96.302 | -2.063 | No | No | No | 0.317 | 0.335 | 0.284 | -2.218 |
|  | 7 | 1,4-Di-tert-butylbenzene | -5.968 | 1.503 | 94.096 | -1.821 | No | No | No | 0.977 | 0 | 0.561 | -0.838 |
|  | 8 | Tridecane | -7.131 | 1.377 | 92.077 | -1.587 | No | No | No | 0.618 | 0.151 | 0.882 | -1.581 |
|  | 9 | beta-Elemene | -6.43 | 1.41 | 94.359 | -1.279 | No | No | No | 0.538 | 0.316 | 0.695 | -2.445 |
|  | 10 | 8-Isopropenyl-1,5-dimethyl-cyclodeca-1,5-diene | -5.412 | 1.424 | 94.928 | -1.587 | No | No | No | 0.601 | 0.157 | 0.809 | -1.714 |
|  | 11 | (+)-Helminthogermacrene | -5.412 | 1.424 | 94.928 | -1.587 | No | No | No | 0.538 | 0.316 | 0.695 | -2.445 |
|  | 12 | Tetradecane | -7.528 | 1.376 | 91.733 | -1.859 | No | No | No | 0.646 | 0.11 | 0.901 | -1.526 |
|  | 13 | 2-Isopropenyl-1-methyl-4-(1-methylethylidene)-1-vinylcyclohexane | -6.501 | 1.405 | 93.381 | -1.329 | No | No | No | 0.572 | 0.153 | 0.775 | -1.641 |
|  | 14 | Humulene | -5.191 | 1.421 | 94.682 | -1.739 | Yes | No | No | 0.505 | 0.347 | 0.663 | -2.555 |
|  | 15 | alpha-Longipinene | -5.051 | 1.387 | 95.793 | -1.988 | No | No | No | 0.894 | 0.265 | 0.802 | -2.396 |
|  | 16 | Cedrene | -5.822 | 1.403 | 96.571 | -1.792 | No | No | No | 0.774 | 0.158 | 0.811 | -1.809 |
|  | 17 | gamma-Muurolene | -6.228 | 1.427 | 96.475 | -1.561 | No | No | No | 0.678 | 0.195 | 0.785 | -1.895 |
|  | 18 | 1,2,4a,5,6,8a-Hexahydro-1-isopropyl-4,7-dimethylnaphthalene | -5.92 | 1.415 | 94.64 | -1.443 | No | No | No | 0.67 | 0.126 | 0.809 | -1.631 |
|  | 19 | (-)-Alloaromadendrene | -5.764 | 1.395 | 95.302 | -1.828 | No | No | No | 0.505 | 0.347 | 0.663 | -2.555 |
|  | 20 | beta-Humulene | -5.191 | 1.421 | 94.682 | -1.739 | Yes | No | No | 0.753 | 0.165 | 0.822 | -1.769 |
|  | 21 | 2-Isopropenyl-4a,8-dimethyl-1,2,3,4,4a,5,6,7-octahydronaphthalene | -6.143 | 1.407 | 94.716 | -1.452 | No | No | No | 0.698 | 0.176 | 0.772 | -1.885 |
|  | 22 | Selina-4(15),7(11)-diene | -6.496 | 1.424 | 95.061 | -1.763 | No | No | No | 0.612 | 0.084 | 0.778 | -1.404 |
|  | 23 | 2,5-Di-tert-butylphenol | -4.711 | 1.582 | 91.359 | -2.048 | No | No | No | 0.545 | 0.042 | 0.47 | -0.858 |
|  | 24 | 3,5-Di-tert-butylphenol | -3.876 | 1.668 | 92.254 | -2.364 | No | No | No | 0.735 | 0.009 | 0.469 | -1.052 |
|  | 25 | (+)-delta-Cadinene | -5.915 | 1.422 | 96.128 | -1.462 | No | No | No | 0.689 | 0.196 | 0.773 | -1.945 |
|  | 26 | (+)-alpha-Muurolene | -5.92 | 1.415 | 94.64 | -1.443 | No | No | No | 0.678 | 0.195 | 0.785 | -1.895 |
|  | 27 | 4a,5-Dimethyl-3-(prop-1-en-2-yl)-1,2,3,4,4a,5,6,7-octahydronaphthalene | -6.074 | 1.401 | 94.127 | -1.461 | No | No | No | 0.686 | 0.186 | 0.776 | -1.865 |
|  | 28 | (+)-Aromadendrene | -5.764 | 1.395 | 95.302 | -1.828 | No | No | No | 0.747 | 0.107 | 0.946 | -1.422 |
|  | 29 | (+)-Cyclosativene | -5.343 | 1.36 | 95.698 | -2.526 | No | No | No | 0.753 | 0.165 | 0.822 | -1.769 |
|  | 30 | 3,7(11)-Eudesmadiene | -6.081 | 1.42 | 94.949 | -1.496 | No | No | No | 0.67 | 0.177 | 0.754 | -1.852 |
|  | 31 | Epizonarene | -5.925 | 1.422 | 95.431 | -1.451 | No | No | No | 0.684 | 0.196 | 0.773 | -1.935 |
|  | 32 | (-)-alpha-Gurjunene | -5.427 | 1.41 | 96.566 | -1.746 | No | No | No | 0.786 | 0.231 | 0.792 | -2.136 |
|  | 33 | Patchoulene | -5.734 | 1.4 | 95.658 | -1.73 | No | No | No | 0.786 | 0.188 | 0.791 | -1.959 |
|  | 34 | 1H-Benzocyclohepten-7-ol, 2,3,4,4a,5,6,7,8-octahydro-1,1,4a,7-tetramethyl-, cis- | -4.366 | 1.498 | 91.951 | -1.946 | No | No | No | 0.462 | 0.304 | 0.553 | -2.399 |
|  | 35 | Patchoulane | -6.093 | 1.399 | 96.014 | -2.065 | No | No | No | 0.696 | 0.091 | 0.84 | -1.488 |
|  | 36 | 3,4-Dimethyl-3-cyclohexenylmethanal | -2.241 | 1.504 | 96.67 | -2.15 | Yes | No | No | 0.176 | 0.543 | 0.586 | -2.408 |
|  | 37 | (-)-gamma-Cadinene | -6.228 | 1.427 | 96.475 | -1.561 | No | No | No | 0.67 | 0.126 | 0.809 | -1.631 |
|  | 38 | alpha-Cadinene | -5.92 | 1.415 | 94.64 | -1.443 | No | No | No | 0.678 | 0.195 | 0.785 | -1.895 |
|  | 39 | 7R,8R-8-Hydroxy-4-isopropylidene-7-methylbicyclo [5.3.1]undec-1-ene | -3.929 | 1.507 | 93.086 | -2.205 | No | No | No | 0.445 | 0.38 | 0.535 | -2.766 |
|  | 40 | Octadecane | -8.481 | 1.373 | 90.358 | -2.644 | No | No | No | 0.661 | 0 | 0.977 | -1.308 |
|  | 41 | (1E,5E,11E)-1,5,11-Trimethyl-8-isopropenylcyclotetradeca-1,5,11-triene | -6.943 | 1.445 | 93.234 | -1.767 | No | No | No | 0.663 | 0.164 | 0.664 | -2.513 |
|  | 42 | Eicosane | -8.59 | 1.371 | 89.671 | -2.774 | No | No | Yes | 0.614 | 0 | 1.014 | -1.199 |
|  | 43 | Bis(2-ethylhexyl) phthalate | -6.47 | 1.408 | 92.45 | -2.67 | No | Yes | Yes | 0.36 | 0 | -0.175 | -2.213 |
|  | 44 | Diisooctyl phthalate | -6.757 | 1.425 | 91.448 | -2.656 | No | Yes | Yes | 0.194 | 0 | -0.184 | -2.169 |
|  | 45 | Phthalic acid, di(2-propylphenyl) ester | -6.397 | 1.124 | 95.02 | -2.734 | No | Yes | Yes | -0.665 | 0.053 | -0.09 | -1.792 |
|  | 46 | 2-Amino-3,5-dibromopyridine | -2.403 | 1.533 | 91.851 | -3.109 | No | No | No | -0.109 | 0.546 | 0.268 | -2.629 |
| Methanol Extract | 47 | Succinic acid, hex-4-yn-3-yl pentyl ester | -2.819 | 1.555 | 96.383 | -2.253 | No | No | No | -0.098 | 0.369 | -0.052 | -2.801 |
|  | 48 | Methyl palmitate | -6.927 | 1.6 | 92.335 | -2.595 | No | No | No | 0.334 | 0.074 | 0.749 | -1.678 |
|  | 49 | Palmitic cid | -5.562 | 1.558 | 92.004 | -2.717 | No | No | No | -0.543 | 0.101 | -0.111 | -1.816 |
|  | 50 | Methyl elaidate | -7.436 | 1.605 | 92.154 | -2.758 | No | No | Yes | 0.299 | 0.027 | 0.777 | -1.516 |
|  | 51 | Methyl oleate | -7.436 | 1.605 | 92.154 | -2.758 | No | No | Yes | 0.299 | 0.027 | 0.777 | -1.516 |
|  | 52 | 11-Octadecenoic acid methyl ester | -7.436 | 1.605 | 92.154 | -2.758 | No | No | Yes | 0.299 | 0.027 | 0.777 | -1.516 |
|  | 53 | Methyl stearate | -7.51 | 1.598 | 91.648 | -2.792 | No | No | Yes | 0.325 | 0.027 | 0.787 | -1.569 |
|  | 54 | Oleic acid | -5.924 | 1.563 | 91.823 | -2.725 | No | No | No | -0.558 | 0.052 | -0.168 | -1.654 |
|  | 55 | 9-Octadecenoic acid | -5.924 | 1.563 | 91.823 | -2.725 | No | No | No | -0.558 | 0.052 | -0.168 | -1.654 |
|  | 56 | Elaidic acid | -5.924 | 1.563 | 91.823 | -2.725 | No | No | No | -0.558 | 0.052 | -0.168 | -1.654 |
|  | 57 | Stigmastan-3,5-diene | -7.256 | 1.227 | 96.148 | -2.732 | No | No | Yes | 0.391 | 0 | 0.96 | -1.507 |
|  | 58 | Podocarpan-14-ol | -5.244 | 1.492 | 93.103 | -2.347 | No | No | No | 0.554 | 0.128 | 0.63 | -1.984 |

**Additional File: Table 3b: Metabolism and Excretion properties of the identified compounds using pkCSM tool**

| S.No. | | Compound Name | | | | | | | Metabolism | | | | | | | Excretion | |
| --- | --- | --- | --- | --- | --- | --- | --- | --- | --- | --- | --- | --- | --- | --- | --- | --- | --- |
|  |  |  |  |  |  |  |  |  | CYP2D6  substrate | CYP3A4  substrate | CYP1A2  inhibitor | CYP2C19  inhibitor | CYP2C9  inhibitor | CYP2D6  inhibitor | CYP3A4  inhibitior | Total  Clearance | Renal OCT2 substrate |
| 1 | | 1,3-Dichloropropane | | | | | | | No | No | No | No | No | No | No | 0.42 | No |
| 2 | | 5-Indanol | | | | | | | No | No | Yes | No | No | No | No | 0.12 | No |
| 3 | | Terephthalaldehyde | | | | | | | No | No | No | No | No | No | No | 0.22 | No |
| 4 | | Isophthalaldehyde | | | | | | | No | No | No | No | No | No | No | 0.24 | No |
| 5 | | 1,3-Di-tert-butylbenzene | | | | | | | No | Yes | Yes | No | No | No | No | 1.08 | No |
| 6 | | Precocene I | | | | | | | No | No | Yes | No | No | No | No | 0.29 | No |
| 7 | | 1,4-Di-tert-butylbenzene | | | | | | | No | Yes | Yes | No | No | No | No | 1.07 | No |
| 8 | | Tridecane | | | | | | | No | No | No | No | No | No | No | 1.73 | No |
| 9 | | beta-Elemene | | | | | | | No | No | No | No | No | No | No | 1.44 | No |
| 10 | | 8-Isopropenyl-1,5-dimethyl-cyclodeca-1,5-diene | | | | | | | No | No | No | No | No | No | No | 0.25 | No |
| 11 | | (+)-Helminthogermacrene | | | | | | | No | No | No | No | No | No | No | 1.44 | No |
| 12 | | Tetradecane | | | | | | | No | No | No | No | No | No | No | 1.77 | No |
| 13 | | 2-Isopropenyl-1-methyl-4-(1-methylethylidene)-1-vinylcyclohexane | | | | | | | No | Yes | No | No | No | No | No | 1.41 | No |
| 14 | | Humulene | | | | | | | No | No | No | No | No | No | No | 1.28 | No |
| 15 | | alpha-Longipinene | | | | | | | No | No | No | No | Yes | No | No | 0.86 | No |
| 16 | | Cedrene | | | | | | | No | Yes | No | No | No | No | No | 0.93 | No |
| 17 | | gamma-Muurolene | | | | | | | No | No | No | No | No | No | No | 1.18 | No |
| 18 | | 1,2,4a,5,6,8a-Hexahydro-1-isopropyl-4,7-dimethylnaphthalene | | | | | | | No | No | No | No | No | No | No | 1.19 | No |
| 19 | | (-)-Alloaromadendrene | | | | | | | No | No | No | No | No | No | No | 1.29 | No |
| 20 | | beta-Humulene | | | | | | | No | Yes | No | No | No | No | No | 0.93 | No |
| 21 | | 2-Isopropenyl-4a,8-dimethyl-1,2,3,4,4a,5,6,7-octahydronaphthalene | | | | | | | No | No | No | No | No | No | No | 1.17 | No |
| 22 | | Selina-4(15),7(11)-diene | | | | | | | No | Yes | Yes | No | No | No | No | 1.13 | No |
| 23 | | 2,5-Di-tert-butylphenol | | | | | | | No | Yes | Yes | No | No | No | No | 0.78 | No |
| 24 | | 3,5-Di-tert-butylphenol | | | | | | | No | Yes | Yes | No | No | No | No | 0.75 | No |
| 25 | | (+)-delta-Cadinene | | | | | | | No | No | No | No | No | No | No | 1.18 | No |
| 26 | | (+)-alpha-Muurolene | | | | | | | No | No | No | No | No | No | No | 1.18 | No |
| 27 | | 4a,5-Dimethyl-3-(prop-1-en-2-yl)-1,2,3,4,4a,5,6,7-octahydronaphthalene | | | | | | | No | Yes | No | No | No | No | No | 1.21 | No |
| 28 | | (+)-Aromadendrene | | | | | | | No | Yes | Yes | No | No | No | No | 0.77 | No |
| 29 | | (+)-Cyclosativene | | | | | | | No | Yes | No | No | No | No | No | 0.93 | No |
| 30 | | 3,7(11)-Eudesmadiene | | | | | | | No | No | No | No | No | No | No | 1.13 | No |
| 31 | | Epizonarene | | | | | | | No | No | No | No | No | No | No | 1.18 | No |
| 32 | | (-)-alpha-Gurjunene | | | | | | | No | No | No | No | Yes | No | No | 0.91 | No |
| 33 | | Patchoulene | | | | | | | No | Yes | No | No | No | No | No | 0.94 | No |
| 34 | | 1H-Benzocyclohepten-7-ol, 2,3,4,4a,5,6,7,8-octahydro-1,1,4a,7-tetramethyl-, cis- | | | | | | | No | No | No | Yes | Yes | No | No | 0.93 | No |
| 35 | | Patchoulane | | | | | | | No | Yes | Yes | No | No | No | No | 0.91 | No |
| 36 | | 3,4-Dimethyl-3-cyclohexenylmethanal | | | | | | | No | No | No | No | No | No | No | 0.26 | No |
| 37 | | (-)-gamma-Cadinene | | | | | | | No | No | No | No | No | No | No | 1.19 | No |
| 38 | | alpha-Cadinene | | | | | | | No | No | No | No | No | No | No | 1.18 | No |
| 39 | | 7R,8R-8-Hydroxy-4-isopropylidene-7-methylbicyclo[5.3.1]undec-1-ene | | | | | | | No | No | No | Yes | No | No | No | 1.16 | No |
| 40 | | Octadecane | | | | | | | No | Yes | Yes | No | No | No | No | 1.92 | No |
| 41 | | (1E,5E,11E)-1,5,11-Trimethyl-8-isopropenylcyclotetradeca-1,5,11-triene | | | | | | | No | No | No | Yes | No | No | No | 1.51 | Yes |
| 42 | | Eicosane | | | | | | | No | Yes | Yes | No | No | No | No | 2 | No |
| 43 | | Bis(2-ethylhexyl) phthalate | | | | | | | No | Yes | No | Yes | No | No | No | 1.9 | No |
| 44 | | Diisooctyl phthalate | | | | | | | No | Yes | No | No | No | No | No | 1.65 | No |
| 45 | | Phthalic acid, di(2-propylphenyl) ester | | | | | | | No | Yes | No | Yes | Yes | No | Yes | 0.82 | No |
| 46 | | 2-Amino-3,5-dibromopyridine | | | | | | | No | No | Yes | No | No | No | No | -0.14 | No |
| 47 | | Succinic acid, hex-4-yn-3-yl pentyl ester | | | | | | | No | No | No | No | No | No | No | 1.82 | No |
| 48 | | Methyl palmitate | | | | | | | No | Yes | Yes | No | No | No | No | 1.86 | No |
| 49 | | Palmitic acid | | | | | | | No | Yes | No | No | No | No | No | 1.76 | No |
| 50 | | Methyl elaidate | | | | | | | No | Yes | Yes | No | No | No | No | 1.98 | No |
| 51 | | Methyl oleate | | | | | | | No | Yes | Yes | No | No | No | No | 1.98 | No |
| 52 | | 11-Octadecenoic acid methyl ester | | | | | | | No | Yes | Yes | No | No | No | No | 1.98 | No |
| 53 | | Methyl stearate | | | | | | | No | Yes | Yes | No | No | No | No | 1.93 | No |
| 54 | | Oleic acid | | | | | | | No | Yes | Yes | No | No | No | No | 1.88 | No |
| 55 | | 9-Octadecenoic acid | | | | | | | No | Yes | Yes | No | No | No | No | 1.88 | No |
| 56 | | Elaidic acid | | | | | | | No | Yes | Yes | No | No | No | No | 1.88 | No |
| 57 | | Stigmastan-3,5-diene | | | | | | | No | Yes | No | No | No | No | No | 0.63 | No |
| 58 | | Podocarpan-14-ol | | | | | | | No | Yes | Yes | Yes | No | No | No | 0.88 | No |
|  | |  | |  | |  | | |  |  |  |  |  |  |  |  |  |
|  | |  | |  |  |  |  |  |  |  |  |  |  |  |  |  |  |
|  | |  |  |  |  |  |  |  |  |  |  |  |  |  |  |  |  |
|  | |  |  |  |  |  |  |  |  |  |  |  |  |  |  |  |  |
|  |  | |  | |  | |  |  |  |  |  |  |  |  |  |  |  |

**Additional File: Table 4_Toxicity properties of the identified compounds using pkCSM tool**

| S.No. | Compound Name | Toxicity | | | | | | | | | |
| --- | --- | --- | --- | --- | --- | --- | --- | --- | --- | --- | --- |
|  |  | AMES toxicity | Max. tolerated  dose (human) | hERG I inhibitor | hERG II inhibitor | Oral Rat Acute  Toxicity (LD50) | Oral Rat Chronic  Toxicity (LOAEL) | Hepatotoxicity | Skin Sensitisation | *T.Pyriformis*  toxicity | Minnow toxicity |
| 1 | 1,3-Dichloropropane | Yes | 0.99 | No | No | 2.41 | 1.703 | No | No | 0.627 | 1.32 |
| 2 | 5-Indanol | No | 0.34 | No | No | 2.05 | 1.976 | Yes | Yes | 0.658 | 1.45 |
| 3 | Terephthalaldehyde | No | 0.97 | No | No | 1.79 | 2.606 | No | Yes | -0.2 | 1.86 |
| 4 | Isophthalaldehyde | No | 0.97 | No | No | 1.77 | 2.606 | No | Yes | -0.2 | 1.86 |
| 5 | 1,3-Di-tert-butylbenzene | No | 0.39 | No | No | 1.93 | 1.535 | No | Yes | 1.345 | -0.22 |
| 6 | Precocene I | No | 0.77 | No | No | 1.84 | 2.086 | No | Yes | 1.165 | 0.77 |
| 7 | 1,4-Di-tert-butylbenzene | No | 0.44 | No | No | 1.89 | 1.491 | No | Yes | 1.393 | -0.23 |
| 8 | Tridecane | No | 0.27 | No | No | 1.54 | 1.413 | No | Yes | 2.044 | -0.67 |
| 9 | beta-Elemene | No | 0.06 | No | No | 1.54 | 1.309 | No | Yes | 1.901 | 0.12 |
| 10 | 8-Isopropenyl-1,5-dimethyl-cyclodeca-1,5-diene | No | 0.54 | No | No | 1.76 | 1.347 | No | Yes | 1.572 | 0.5 |
| 11 | (+)-Helminthogermacrene | No | 0.54 | No | No | 1.76 | 1.347 | No | Yes | 1.572 | 0.5 |
| 12 | Tetradecane | No | 0.22 | No | No | 1.53 | 1.377 | No | Yes | 2.07 | -0.93 |
| 13 | 2-Isopropenyl-1-methyl-4-(1-methylethylidene)-1-vinylcyclohexane | No | -0.08 | No | No | 1.51 | 1.338 | No | Yes | 1.771 | 0.22 |
| 14 | Humulene | No | 0.55 | No | No | 1.77 | 1.336 | No | Yes | 1.451 | 0.72 |
| 15 | alpha-Longipinene | No | 0.14 | No | No | 1.68 | 1.334 | No | No | 0.909 | 0.56 |
| 16 | Cedrene | No | -0.07 | No | No | 1.56 | 1.399 | No | No | 1.43 | 0.19 |
| 17 | gamma-Muurolene | No | 0.05 | No | No | 1.54 | 1.473 | No | Yes | 1.73 | -0.02 |
| 18 | 1,2,4a,5,6,8a-Hexahydro-1-isopropyl-4,7-dimethylnaphthalene | No | 0.13 | No | No | 1.53 | 1.382 | No | Yes | 1.622 | 0.37 |
| 19 | (-)-Alloaromadendrene | No | -0.15 | No | No | 1.53 | 1.332 | No | No | 1.431 | 0.43 |
| 20 | beta-Humulene | No | 0.55 | No | No | 1.77 | 1.336 | No | Yes | 1.451 | 0.72 |
| 21 | 2-Isopropenyl-4a,8-dimethyl-1,2,3,4,4a,5,6,7-octahydronaphthalene | No | 0.05 | No | No | 1.59 | 1.385 | No | Yes | 1.624 | 0.2 |
| 22 | Selina-4(15),7(11)-diene | No | -0.2 | No | No | 1.57 | 1.557 | No | Yes | 1.65 | -0.02 |
| 23 | 2,5-Di-tert-butylphenol | No | 0.54 | No | No | 2.14 | 1.44 | No | Yes | 1.493 | 0.11 |
| 24 | 3,5-Di-tert-butylphenol | No | 0.41 | No | No | 2.35 | 1.736 | No | Yes | 1.667 | -0.11 |
| 25 | (+)-delta-Cadinene | No | 0.21 | No | No | 1.55 | 1.448 | No | Yes | 1.61 | 0.09 |
| 26 | (+)-alpha-Muurolene | No | 0.13 | No | No | 1.53 | 1.382 | No | Yes | 1.622 | 0.37 |
| 27 | 4a,5-Dimethyl-3-(prop-1-en-2-yl)-1,2,3,4,4a,5,6,7-octahydronaphthalene | No | -0.02 | No | No | 1.54 | 1.351 | No | Yes | 1.623 | 0.35 |
| 28 | (+)-Aromadendrene | No | -0.15 | No | No | 1.53 | 1.332 | No | No | 1.431 | 0.43 |
| 29 | (+)-Cyclosativene | No | -0.32 | No | No | 1.69 | 1.366 | No | No | 0.681 | 0.16 |
| 30 | 3,7(11)-Eudesmadiene | No | 0.14 | No | No | 1.57 | 1.436 | No | Yes | 1.54 | 0.22 |
| 31 | Epizonarene | No | 0.21 | No | No | 1.55 | 1.448 | No | Yes | 1.61 | 0.18 |
| 32 | (-)-alpha-Gurjunene | No | 0.21 | No | No | 1.61 | 1.371 | No | No | 1.263 | 0.36 |
| 33 | Patchoulene | No | 0 | No | No | 1.57 | 1.387 | No | No | 1.362 | 0.31 |
| 34 | 1H-Benzocyclohepten-7-ol, 2,3,4,4a,5,6,7,8-octahydro-1,1,4a,7-tetramethyl-, cis- | No | 0.16 | No | No | 1.69 | 1.238 | No | Yes | 1.343 | 1.03 |
| 35 | Patchoulane | No | -0.32 | No | No | 1.64 | 1.402 | No | No | 1.415 | 0.07 |
| 36 | 3,4-Dimethyl-3-cyclohexenylmethanal | No | 0.77 | No | No | 1.82 | 1.994 | No | Yes | 0.067 | 1.56 |
| 37 | (-)-gamma-Cadinene | No | 0.05 | No | No | 1.54 | 1.473 | No | Yes | 1.73 | -0.02 |
| 38 | alpha-Cadinene | No | 0.13 | No | No | 1.53 | 1.382 | No | Yes | 1.622 | 0.37 |
| 39 | 7R,8R-8-Hydroxy-4-isopropylidene-7-methylbicyclo[5.3.1]undec-1-ene | No | 0.47 | No | No | 1.8 | 1.242 | No | Yes | 1.088 | 1.18 |
| 40 | Octadecane | No | 0.07 | No | Yes | 1.54 | 1.241 | No | Yes | 1.37 | -1.89 |
| 41 | (1E,5E,11E)-1,5,11-Trimethyl-8-isopropenylcyclotetradeca-1,5,11-triene | No | 0.32 | No | No | 1.61 | 1.161 | No | Yes | 1.913 | -0.13 |
| 42 | Eicosane | No | -0.01 | No | Yes | 1.59 | 1.177 | No | Yes | 0.926 | -2.37 |
| 43 | Bis(2-ethylhexyl) phthalate | No | 1.39 | No | Yes | 1.45 | 2.535 | No | No | 0.779 | -2.27 |
| 44 | Diisooctyl phthalate | No | 1.11 | No | Yes | 1.25 | 2.695 | No | No | 0.664 | -3.43 |
| 45 | Phthalic acid, di(2-propylphenyl) ester | No | 0.6 | No | Yes | 2.32 | 2.281 | Yes | No | 0.295 | -2.02 |
| 46 | 2-Amino-3,5-dibromopyridine | No | 1.04 | No | No | 2.29 | 1.391 | No | Yes | 0.431 | 1.53 |
| 47 | Succinic acid, hex-4-yn-3-yl pentyl ester | No | 0.81 | No | No | 1.694 | 1.889 | No | No | 0.951 | 0.597 |
| 48 | Methyl palmitate | No | 0.178 | No | No | 1.635 | 2.998 | No | Yes | 1.935 | -1.37 |
| 49 | Palmitic acid | No | -0.71 | No | No | 1.44 | 3.181 | No | Yes | 0.84 | -1.08 |
| 50 | Methyl elaidate | No | 0.04 | No | No | 1.637 | 3.075 | No | Yes | 1.529 | -1.73 |
| 51 | Methyl oleate | No | 0.04 | No | No | 1.637 | 3.075 | No | Yes | 1.529 | -1.73 |
| 52 | 11-Octadecenoic acid methyl ester | No | 0.04 | No | No | 1.637 | 3.075 | No | Yes | 1.529 | -1.73 |
| 53 | Methyl stearate | No | 0.099 | No | No | 1.656 | 3.147 | No | Yes | 1.448 | -1.85 |
| 54 | Oleic acid | No | -0.81 | No | No | 1.417 | 3.259 | No | Yes | 0.676 | -1.44 |
| 55 | 9-Octadecenoic acid | No | -0.81 | No | No | 1.417 | 3.259 | No | Yes | 0.676 | -1.44 |
| 56 | Elaidic acid | No | -0.81 | No | No | 1.417 | 3.259 | No | Yes | 0.676 | -1.44 |
| 57 | Stigmastan-3,5-diene | No | -0.28 | No | Yes | 2.185 | 0.962 | No | No | 0.418 | -2.49 |
| 58 | Podocarpan-14-ol | No | -0.52 | No | No | 1.731 | 1.174 | No | Yes | 1.614 | 0.461 |

**Additional File: Table 5_ADME properties of the identified compounds using SwissADME tool**

|  | Absorption | | | | | Distribution | | Metabolism | | | | |
| --- | --- | --- | --- | --- | --- | --- | --- | --- | --- | --- | --- | --- |
| Compound Name | Consensus Log P | Ali Class | GI absorption | log Kp (cm/s) | Pgp substrate | BBB permeant | Bioavailability Score | CYP1A2 inhibitor | CYP2C19 inhibitor | CYP2C9 inhibitor | CYP2D6 inhibitor | CYP3A4 inhibitor |
| 1,3-Dichloropropane | 1.93 | Very soluble | Low | -5.57 | No | No | 0.55 | No | No | No | No | No |
| Isophthalaldehyde | 1.26 | Very soluble | High | -6.33 | No | Yes | 0.55 | Yes | No | No | No | No |
| Terephthalaldehyde | 1.23 | Very soluble | High | -6.46 | No | Yes | 0.55 | Yes | No | No | No | No |
| 5-Indanol | 2.15 | Soluble | High | -5.44 | No | Yes | 0.55 | No | No | No | No | No |
| 3,4-Dimethyl-3-cyclohexenylmethanal | 1.97 | Very soluble | High | -6.4 | No | Yes | 0.55 | No | No | No | No | No |
| Tridecane | 5.31 | Poorly soluble | Low | -2.7 | No | No | 0.55 | Yes | No | No | No | No |
| Precocene I | 2.77 | Soluble | High | -5.08 | No | Yes | 0.55 | Yes | No | No | Yes | No |
| 1,4-Di-tert-butylbenzene | 4.77 | Poorly soluble | Low | -2.86 | No | Yes | 0.55 | No | No | No | Yes | No |
| 1,3-Di-tert-butylbenzene | 4.63 | Moderately soluble | Low | -3.36 | No | Yes | 0.55 | No | No | No | Yes | No |
| Tetradecane | 5.68 | Poorly soluble | Low | -2.4 | No | No | 0.55 | Yes | No | No | No | No |
| Humulene | 4.26 | Moderately soluble | Low | -4.32 | No | No | 0.55 | No | No | Yes | No | No |
| beta-Patchoulene | 4.4 | Moderately soluble | Low | -4.5 | No | No | 0.55 | No | Yes | Yes | No | No |
| 4a,5-Dimethyl-3-(prop-1-en-2-yl)-1,2,3,4,4a,5,6,7-octahydronaphthalene | 4.41 | Moderately soluble | Low | -3.83 | No | No | 0.55 | No | Yes | Yes | No | No |
| Cedrene | 4.36 | Moderately soluble | Low | -4.27 | No | No | 0.55 | No | Yes | Yes | No | No |
| (-)-alpha-Gurjunene | 4.27 | Soluble | Low | -4.64 | No | No | 0.55 | No | Yes | Yes | No | No |
| (+)-Aromadendrene | 4.34 | Moderately soluble | Low | -4.2 | No | Yes | 0.55 | Yes | Yes | Yes | No | No |
| (+)-alpha-Longipinene | 4.37 | Moderately soluble | Low | -4.27 | No | No | 0.55 | No | Yes | Yes | No | No |
| (+)-gamma-Cadinene | 4.18 | Moderately soluble | Low | -4.49 | No | No | 0.55 | No | Yes | Yes | No | No |
| alpha-Cadinene | 4.08 | Soluble | Low | -4.65 | No | No | 0.55 | No | Yes | Yes | No | No |
| Aromadendrene | 4.34 | Moderately soluble | Low | -4.2 | No | Yes | 0.55 | Yes | Yes | Yes | No | No |
| beta-Humulene | 4.37 | Moderately soluble | Low | -4.15 | No | No | 0.55 | No | No | Yes | No | No |
| Naphthalene, 1,2,3,4,4a,5,6,8a-octahydro-7-methyl-4-methylene-1-(1-methylethyl)-, (1alpha,4aalpha,8aalpha)- | 4.18 | Moderately soluble | Low | -4.49 | No | No | 0.55 | No | Yes | Yes | No | No |
| 3,7(11)-Eudesmadiene | 4.31 | Moderately soluble | Low | -4.39 | No | No | 0.55 | No | Yes | Yes | No | No |
| 1,2,4a,5,6,8a-Hexahydro-1-isopropyl-4,7-dimethylnaphthalene | 4.08 | Soluble | Low | -4.65 | No | No | 0.55 | No | Yes | Yes | No | No |
| Cadina-1(10),4-diene | 4.12 | Soluble | Low | -4.85 | No | No | 0.55 | No | Yes | Yes | No | No |
| (-)-cis-beta-Elemene | 4.63 | Moderately soluble | Low | -3.21 | No | No | 0.55 | No | Yes | Yes | No | No |
| alpha-Bergamotene | 4.7 | Poorly soluble | Low | -2.97 | No | No | 0.55 | No | Yes | Yes | No | No |
| 2-Isopropenyl-1-methyl-4-(1-methylethylidene)-1-vinylcyclohexane | 4.56 | Moderately soluble | Low | -3.75 | No | No | 0.55 | No | Yes | Yes | No | No |
| (+)-Cyclosativene | 4.32 | Moderately soluble | Low | -4.09 | No | Yes | 0.55 | Yes | Yes | Yes | No | No |
| Epizonarene | 4.18 | Soluble | Low | -4.69 | No | No | 0.55 | No | Yes | Yes | No | No |
| (+)-alpha-Muurolene | 4.08 | Soluble | Low | -4.65 | No | No | 0.55 | No | Yes | Yes | No | No |
| 2-Isopropenyl-4a,8-dimethyl-1,2,3,4,4a,5,6,7-octahydronaphthalene | 4.46 | Moderately soluble | Low | -4.06 | No | No | 0.55 | No | Yes | Yes | No | No |
| Eudesma-4(14),7(11)-diene | 4.39 | Moderately soluble | Low | -4.22 | No | No | 0.55 | No | Yes | Yes | No | No |
| (+)-Helminthogermacrene | 4.39 | Moderately soluble | Low | -4.22 | No | No | 0.55 | No | Yes | Yes | No | No |
| Naphthalene, 1,2,3,4,4a,5,6,8a-octahydro-4a,8-dimethyl-2-(1-methylethenyl)-, [2R-(2alpha,4aalpha,8abeta)]- | 4.4 | Moderately soluble | Low | -3.85 | No | No | 0.55 | No | Yes | Yes | No | No |
| 8-Isopropenyl-1,5-dimethyl-cyclodeca-1,5-diene | 4.4 | Moderately soluble | Low | -3.85 | No | No | 0.55 | No | Yes | Yes | No | No |
| 3,5-Di-tert-butylphenol | 3.89 | Moderately soluble | High | -4.07 | No | Yes | 0.55 | No | No | No | Yes | No |
| Patchoulane | 4.62 | Moderately soluble | Low | -3.54 | No | No | 0.55 | Yes | Yes | Yes | No | No |
| 7R,8R-8-Hydroxy-4-isopropylidene-7-methylbicyclo[5.3.1]undec-1-ene | 3.48 | Soluble | High | -5.33 | No | Yes | 0.55 | No | No | Yes | No | No |
| 1H-Benzocyclohepten-7-ol, 2,3,4,4a,5,6,7,8-octahydro-1,1,4a,7-tetramethyl-, cis- | 3.72 | Moderately soluble | High | -4.76 | No | Yes | 0.55 | No | No | Yes | No | No |
| 2-Amino-3,5-dibromopyridine | 1.97 | Soluble | High | -6.49 | No | Yes | 0.55 | Yes | No | No | No | No |
| Octadecane | 7.18 | Poorly soluble | Low | -1.2 | No | No | 0.55 | Yes | No | No | No | No |
| Nonadecane | 7.54 | Poorly soluble | Low | -0.9 | No | No | 0.55 | Yes | No | No | No | No |
| 1,5,9-Cyclotetradecatriene, 1,5,9-trimethyl-12-(1-methylethenyl)- | 5.78 | Poorly soluble | Low | -2.88 | No | No | 0.55 | No | Yes | Yes | No | Yes |
| Eicosane | 7.9 | Insoluble | Low | -0.6 | No | No | 0.55 | Yes | No | No | No | No |
| Bis(2-ethylhexyl) phthalate | 6.17 | Poorly soluble | High | -3.39 | Yes | No | 0.55 | No | No | Yes | No | Yes |
| Diisooctyl phthalate | 6.5 | Poorly soluble | High | -2.71 | No | No | 0.55 | No | No | No | No | No |
| Di-n-2-propylpentylphthalate | 6.16 | Poorly soluble | High | -3.39 | No | No | 0.55 | No | No | No | No | Yes |
| 2,5-Ditert-butyl-4-[[4-[(2,5-ditert-butyl-4-hydroxyphenyl)methyl]piperazin-1-yl]methyl]phenol | 6.92 | Poorly soluble | High | -3.07 | Yes | No | 0.17 | No | No | No | Yes | No |
| Podocarpan-14-ol | 4.2 | Moderately soluble | High | -4.05 | No | Yes | 0.55 | No | Yes | Yes | No | No |
| Palmitic acid | 5.2 | Poorly soluble | High | -2.77 | No | Yes | 0.85 | Yes | No | Yes | No | No |
| Succinic acid, hex-4-yn-3-yl pentyl ester | 3.22 | Soluble | High | -5.65 | No | Yes | 0.55 | No | No | No | No | No |
| Methyl palmitate | 5.54 | Poorly soluble | High | -2.71 | No | Yes | 0.55 | Yes | No | No | No | No |
| Oleic acid | 5.71 | Poorly soluble | High | -2.6 | No | No | 0.85 | Yes | No | Yes | No | No |
| Elaidic acid | 5.65 | Poorly soluble | High | -2.6 | No | No | 0.85 | Yes | No | Yes | No | No |
| 9-Octadecenoic acid | 5.71 | Poorly soluble | High | -2.6 | No | No | 0.85 | Yes | No | Yes | No | No |
| Methyl oleate | 5.95 | Poorly soluble | High | -2.82 | No | No | 0.55 | Yes | No | No | No | No |
| Methyl elaidate | 5.95 | Poorly soluble | High | -2.82 | No | No | 0.55 | Yes | No | No | No | No |
| Methyl vaccenate | 5.92 | Poorly soluble | High | -2.82 | No | No | 0.55 | Yes | No | No | No | No |
| Methyl stearate | 6.24 | Poorly soluble | High | -2.19 | No | No | 0.55 | Yes | No | No | No | No |
| Stigmastan-3,5-diene | 7.95 | Insoluble | Low | -1.22 | No | No | 0.55 | No | No | Yes | No | No |
