# Supplementary material for: GC–MS analysis, molecular docking, and pharmacokinetic studies of Multidentia crassa extracts’ compounds for analgesic and anti-inflammatory activities in dentistry
Source: Sci Rep. 2024 Jan 22;14:1876. doi: 10.1038/s41598-023-47737-x (PMC10803350; doi:10.1038/s41598-023-47737-x)
Supplement: Supplementary file 4 — Supplementary Table 6. [file 41598_2023_47737_MOESM4_ESM.docx]

**Additional File: Table 6a_Comparison of Intestinal Absorption and Distribution properties of the identified compounds using pkCSM and SwissADME tools**

| Compound | Intestinal Absorption | | | Distribution | | | | | |
| --- | --- | --- | --- | --- | --- | --- | --- | --- | --- |
|  | pkCSM(Y/N) | SwissADME (Y/N) | Consensus | pkCSM | | SwissADME | | Consensus | |
|  |  | | | Volume of Distribution | BBB Permeant | Volume of Distribution | BBB Permeant | Volume of Distribution | BBB Permeant |
| 1,3-Dichloropropane | Yes | Yes | Yes | low | Yes | N/A | No | N/A | No |
| Isophthalaldehyde | Yes | Yes | Yes | low | Partially | N/A | Yes | N/A | Yes |
| Terephthalaldehyde | Yes | Yes | Yes | low | Partially | N/A | Yes | N/A | Yes |
| 5-Indanol | Yes | Yes | Yes | High | Yes | N/A | Yes | N/A | Yes |
| 3,4-Dimethyl-3-cyclohexenylmethanal | Yes | Yes | Yes | low | Yes | N/A | Yes | N/A | Yes |
| Tridecane | Yes | Yes | Yes | High | Yes | N/A | No | N/A | No |
| Precocene I | Yes | Yes | Yes | low | Partially | N/A | Yes | N/A | Yes |
| 1,4-Di-tert-butylbenzene | Yes | Yes | Yes | High | Yes | N/A | Yes | N/A | Yes |
| 1,3-Di-tert-butylbenzene | Yes | Yes | Yes | High | Yes | N/A | Yes | N/A | Yes |
| Tetradecane | Yes | Yes | Yes | High | Yes | N/A | No | N/A | No |
| Humulene | Yes | Yes | Yes | High | Yes | N/A | No | N/A | No |
| beta-Patchoulene | Yes | Yes | Yes | High | Yes | N/A | No | N/A | No |
| 4a,5-Dimethyl-3-(prop-1-en-2-yl)-1,2,3,4,4a,5,6,7-octahydronaphthalene | Yes | Yes | Yes | High | Yes | N/A | No | N/A | No |
| Cedrene | Yes | Yes | Yes | High | Yes | N/A | No | N/A | No |
| (-)-alpha-Gurjunene | Yes | Yes | Yes | High | Yes | N/A | No | N/A | No |
| (+)-Aromadendrene | Yes | Yes | Yes | High | Yes | N/A | Yes | N/A | Yes |
| (+)-alpha-Longipinene | Yes | Yes | Yes | High | Yes | N/A | No | N/A | No |
| (+)-gamma-Cadinene | Yes | Yes | Yes | High | Yes | N/A | No | N/A | No |
| alpha-Cadinene | Yes | Yes | Yes | High | Yes | N/A | No | N/A | No |
| Aromadendrene | Yes | Yes | Yes | High | Yes | N/A | Yes | N/A | Yes |
| beta-Humulene | Yes | Yes | Yes | High | Yes | N/A | No | N/A | No |
| Naphthalene, 1,2,3,4,4a,5,6,8a-octahydro-7-methyl-4-methylene-1-(1-methylethyl)-, (1alpha,4aalpha,8aalpha)- | Yes | Yes | Yes | High | Yes | N/A | No | N/A | No |
| 3,7(11)-Eudesmadiene | Yes | Yes | Yes | High | Yes | N/A | No | N/A | No |
| 1,2,4a,5,6,8a-Hexahydro-1-isopropyl-4,7-dimethylnaphthalene | Yes | Yes | Yes | High | Yes | N/A | No | N/A | No |
| Cadina-1(10),4-diene | Yes | Yes | Yes | High | Yes | N/A | No | N/A | No |
| (-)-cis-beta-Elemene | Yes | Yes | Yes | High | Yes | N/A | No | N/A | No |
| alpha-Bergamotene | Yes | Yes | Yes | High | Yes | N/A | No | N/A | No |
| 2-Isopropenyl-1-methyl-4-(1-methylethylidene)-1-vinylcyclohexane | Yes | Yes | Yes | High | Yes | N/A | No | N/A | No |
| (+)-Cyclosativene | Yes | Yes | Yes | High | Yes | N/A | Yes | N/A | Yes |
| Epizonarene | Yes | Yes | Yes | High | Yes | N/A | No | N/A | No |
| (+)-alpha-Muurolene | Yes | Yes | Yes | High | Yes | N/A | No | N/A | No |
| 2-Isopropenyl-4a,8-dimethyl-1,2,3,4,4a,5,6,7-octahydronaphthalene | Yes | Yes | Yes | High | Yes | N/A | No | N/A | No |
| Eudesma-4(14),7(11)-diene | Yes | Yes | Yes | High | Yes | N/A | No | N/A | No |
| (+)-Helminthogermacrene | Yes | Yes | Yes | High | Yes | N/A | No | N/A | No |
| Naphthalene, 1,2,3,4,4a,5,6,8a-octahydro-4a,8-dimethyl-2-(1-methylethenyl)-, [2R-(2alpha,4aalpha,8abeta)]- | Yes | Yes | Yes | High | Yes | N/A | No | N/A | No |
| 8-Isopropenyl-1,5-dimethyl-cyclodeca-1,5-diene | Yes | Yes | Yes | High | Yes | N/A | No | N/A | No |
| 3,5-Di-tert-butylphenol | Yes | Yes | Yes | High | Yes | N/A | Yes | N/A | Yes |
| Patchoulane | Yes | Yes | Yes | High | Yes | N/A | No | N/A | No |
| 7R,8R-8-Hydroxy-4-isopropylidene-7-methylbicyclo[5.3.1]undec-1-ene | Yes | Yes | Yes | low | Yes | N/A | Yes | N/A | Yes |
| 1H-Benzocyclohepten-7-ol, 2,3,4,4a,5,6,7,8-octahydro-1,1,4a,7-tetramethyl-, cis- | Yes | Yes | Yes | High | Yes | N/A | Yes | N/A | Yes |
| 2-Amino-3,5-dibromopyridine | Yes | Yes | Yes | low | Partially | N/A | Yes | N/A | Yes |
| Octadecane | Yes | Yes | Yes | High | Yes | N/A | No | N/A | No |
| Nonadecane | Yes | Yes | Yes | High | Yes | N/A | No | N/A | No |
| 1,5,9-Cyclotetradecatriene, 1,5,9-trimethyl-12-(1-methylethenyl)- | Yes | Yes | Yes | High | Yes | N/A | No | N/A | No |
| Eicosane | Yes | Yes | Yes | High | Yes | N/A | No | N/A | No |
| Bis(2-ethylhexyl) phthalate | Yes | Yes | Yes | low | Partially | N/A | No | N/A | No |
| Diisooctyl phthalate | Yes | Yes | Yes | low | Partially | N/A | No | N/A | No |
| Di-n-2-propylpentylphthalate | Yes | Yes | Yes | low | Partially | N/A | No | N/A | No |
| 2,5-Ditert-butyl-4-[[4-[(2,5-ditert-butyl-4-hydroxyphenyl)methyl]piperazin-1-yl]methyl]phenol | Yes | Yes | Yes | High | Partially | N/A | No | N/A | No |
| Podocarpan-14beta-ol | Yes | Yes | Yes | High | Yes | N/A | Yes | N/A | Yes |
| Palmitic Acid | Yes | Yes | Yes | low | Partially | N/A | Yes | N/A | Yes |
| Succinic acid, hex-4-yn-3-yl pentyl ester | Yes | Yes | Yes | low | Partially | N/A | Yes | N/A | Yes |
| Methyl palmitate | Yes | Yes | Yes | low | Yes | N/A | Yes | N/A | Yes |
| 9-Octadecenoic acid | Yes | Yes | Yes | low | Partially | N/A | No | N/A | No |
| Oleic acid | Yes | Yes | Yes | low | Partially | N/A | No | N/A | No |
| Elaidic acid | Yes | Yes | Yes | low | Partially | N/A | No | N/A | No |
| Methyl elaidate | Yes | Yes | Yes | low | Yes | N/A | No | N/A | No |
| Methyl vaccenate | Yes | Yes | Yes | low | Yes | N/A | No | N/A | No |
| Methyl oleate | Yes | Yes | Yes | low | Yes | N/A | No | N/A | No |
| Methyl stearate | Yes | Yes | Yes | low | Yes | N/A | No | N/A | No |
| Stigmastan-3,5-diene | Yes | Yes | Yes | low | Yes | N/A | No | N/A | No |

**Additional File: Table 6b_Comparison of Metabolism properties of the identified compounds using pkCSM and SwissADME tools**

|  | Metabolism | | | | | | | | | | | | | | | | | |
| --- | --- | --- | --- | --- | --- | --- | --- | --- | --- | --- | --- | --- | --- | --- | --- | --- | --- | --- |
|  | Pgp substrate | | | CYP1A2 inhibitor | | | CYP2C19 inhibitor | | | CYP2C9 inhibitor1 | | | CYP2D6 inhibitor1 | | | CYP3A4 inhibitor | | |
| Compounds | Swissadme | pkcsm | consensus | Swissadme | pkcsm | consensus | Swissadme | pkcsm | consensus | Swissadme | pkcsm | consensus | Swissadme | pkcsm | consensus | Swissadme | pkcsm | consensus |
| 1,3-Dichloropropane | No | Yes | No | No | No | Yes | No | No | Yes | No | No | Yes | No | No | Yes | No | No | Yes |
| Isophthalaldehyde | No | No | Yes | Yes | No | No | No | No | Yes | No | No | Yes | No | No | Yes | No | No | Yes |
| Terephthalaldehyde | No | No | Yes | Yes | No | No | No | No | Yes | No | No | Yes | No | No | Yes | No | No | Yes |
| 5-Indanol | No | No | Yes | No | Yes | No | No | No | Yes | No | No | Yes | No | No | Yes | No | No | Yes |
| 3,4-Dimethyl-3-cyclohexenylmethanal | No | Yes | No | No | No | Yes | No | No | Yes | No | No | Yes | No | No | Yes | No | No | Yes |
| Tridecane | No | No | Yes | Yes | No | No | No | No | Yes | No | No | Yes | No | No | Yes | No | No | Yes |
| Precocene I | No | No | Yes | Yes | Yes | Yes | No | No | Yes | No | No | Yes | Yes | No | No | No | No | Yes |
| 1,4-Di-tert-butylbenzene | No | No | Yes | No | Yes | No | No | No | Yes | No | No | Yes | Yes | No | No | No | No | Yes |
| 1,3-Di-tert-butylbenzene | No | No | Yes | No | Yes | No | No | No | Yes | No | No | Yes | Yes | No | No | No | No | Yes |
| Tetradecane | No | No | Yes | Yes | No | No | No | No | Yes | No | No | Yes | No | No | Yes | No | No | Yes |
| Humulene | No | Yes | No | No | No | Yes | No | No | Yes | Yes | No | No | No | No | Yes | No | No | Yes |
| beta-Patchoulene | No | No | Yes | No | No | Yes | Yes | No | No | Yes | No | No | No | No | Yes | No | No | Yes |
| 4a,5-Dimethyl-3-(prop-1-en-2-yl)-1,2,3,4,4a,5,6,7-octahydronaphthalene | No | No | Yes | No | No | Yes | Yes | No | No | Yes | No | No | No | No | Yes | No | No | Yes |
| Cedrene | No | No | Yes | No | No | Yes | Yes | No | No | Yes | No | No | No | No | Yes | No | No | Yes |
| (-)-alpha-Gurjunene | No | No | Yes | No | No | Yes | Yes | No | No | Yes | Yes | Yes | No | No | Yes | No | No | Yes |
| (+)-Aromadendrene | No | No | Yes | Yes | No | No | Yes | No | No | Yes | No | No | No | No | Yes | No | No | Yes |
| (+)-alpha-Longipinene | No | No | Yes | No | No | Yes | Yes | No | No | Yes | Yes | Yes | No | No | Yes | No | No | Yes |
| (+)-gamma-Cadinene | No | No | Yes | No | No | Yes | Yes | No | No | Yes | No | No | No | No | Yes | No | No | Yes |
| alpha-Cadinene | No | No | Yes | No | No | Yes | Yes | No | No | Yes | No | No | No | No | Yes | No | No | Yes |
| Aromadendrene | No | No | Yes | Yes | No | No | Yes | No | No | Yes | No | No | No | No | Yes | No | No | Yes |
| beta-Humulene | No | Yes | No | No | No | Yes | No | No | Yes | Yes | No | No | No | No | Yes | No | No | Yes |
| Naphthalene, 1,2,3,4,4a,5,6,8a-octahydro-7-methyl-4-methylene-1-(1-methylethyl)-, (1alpha,4aalpha,8aalpha)- | No | No | Yes | No | No | Yes | Yes | No | No | Yes | No | No | No | No | Yes | No | No | Yes |
| 3,7(11)-Eudesmadiene | No | No | Yes | No | No | Yes | Yes | No | No | Yes | No | No | No | No | Yes | No | No | Yes |
| 1,2,4a,5,6,8a-Hexahydro-1-isopropyl-4,7-dimethylnaphthalene | No | No | Yes | No | No | Yes | Yes | No | No | Yes | No | No | No | No | Yes | No | No | Yes |
| Cadina-1(10),4-diene | No | No | Yes | No | No | Yes | Yes | No | No | Yes | No | No | No | No | Yes | No | No | Yes |
| (-)-cis-beta-Elemene | No | No | Yes | No | No | Yes | Yes | No | No | Yes | No | No | No | No | Yes | No | No | Yes |
| alpha-Bergamotene | No | No | Yes | No | No | Yes | Yes | No | No | Yes | No | No | No | No | Yes | No | No | Yes |
| 2-Isopropenyl-1-methyl-4-(1-methylethylidene)-1-vinylcyclohexane | No | No | Yes | No | No | Yes | Yes | No | No | Yes | No | No | No | No | Yes | No | No | Yes |
| (+)-Cyclosativene | No | No | Yes | Yes | Yes | Yes | Yes | No | No | Yes | No | No | No | No | Yes | No | No | Yes |
| Epizonarene | No | No | Yes | No | No | Yes | Yes | No | No | Yes | No | No | No | No | Yes | No | No | Yes |
| (+)-alpha-Muurolene | No | No | Yes | No | No | Yes | Yes | No | No | Yes | No | No | No | No | Yes | No | No | Yes |
| 2-Isopropenyl-4a,8-dimethyl-1,2,3,4,4a,5,6,7-octahydronaphthalene | No | No | Yes | No | No | Yes | Yes | No | No | Yes | No | No | No | No | Yes | No | No | Yes |
| Eudesma-4(14),7(11)-diene | No | No | Yes | No | Yes | No | Yes | No | No | Yes | No | No | No | No | Yes | No | No | Yes |
| (+)-Helminthogermacrene | No | No | Yes | No | No | Yes | Yes | No | No | Yes | No | No | No | No | Yes | No | No | Yes |
| Naphthalene, 1,2,3,4,4a,5,6,8a-octahydro-4a,8-dimethyl-2-(1-methylethenyl)-, [2R-(2alpha,4aalpha,8abeta)]- | No | No | Yes | No | No | Yes | Yes | No | No | Yes | No | No | No | No | Yes | No | No | Yes |
| 8-Isopropenyl-1,5-dimethyl-cyclodeca-1,5-diene | No | No | Yes | No | No | Yes | Yes | No | No | Yes | No | No | No | No | Yes | No | No | Yes |
| 3,5-Di-tert-butylphenol | No | No | Yes | No | Yes | No | No | No | Yes | No | No | Yes | Yes | No | No | No | No | Yes |
| Patchoulane | No | No | Yes | Yes | Yes | Yes | Yes | No | No | Yes | No | No | No | No | Yes | No | No | Yes |
| 7R,8R-8-Hydroxy-4-isopropylidene-7-methylbicyclo[5.3.1]undec-1-ene | No | No | Yes | No | No | Yes | No | Yes | No | Yes | No | No | No | No | Yes | No | No | Yes |
| 1H-Benzocyclohepten-7-ol, 2,3,4,4a,5,6,7,8-octahydro-1,1,4a,7-tetramethyl-, cis- | No | No | Yes | No | No | Yes | No | Yes | No | Yes | Yes | Yes | No | No | Yes | No | No | Yes |
| 2-Amino-3,5-dibromopyridine | No | No | Yes | Yes | Yes | Yes | No | No | Yes | No | No | Yes | No | No | Yes | No | No | Yes |
| Octadecane | No | No | Yes | Yes | Yes | Yes | No | No | Yes | No | No | Yes | No | No | Yes | No | No | Yes |
| Nonadecane | No | No | Yes | Yes | Yes | Yes | No | No | Yes | No | No | Yes | No | No | Yes | No | No | Yes |
| 1,5,9-Cyclotetradecatriene, 1,5,9-trimethyl-12-(1-methylethenyl)- | No | No | Yes | No | No | Yes | Yes | Yes | Yes | Yes | No | No | No | No | Yes | Yes | No | No |
| Eicosane | No | No | Yes | Yes | Yes | Yes | No | No | Yes | No | No | Yes | No | No | Yes | No | No | Yes |
| Bis(2-ethylhexyl) phthalate | Yes | No | No | No | No | Yes | No | Yes | No | Yes | No | No | No | No | Yes | Yes | No | No |
| Diisooctyl phthalate | No | No | Yes | No | No | Yes | No | No | Yes | No | No | Yes | No | No | Yes | No | No | Yes |
| Di-n-2-propylpentylphthalate | No | No | Yes | No | No | Yes | No | Yes | No | No | No | Yes | No | No | Yes | Yes | No | No |
| 2,5-Ditert-butyl-4-[[4-[(2,5-ditert-butyl-4-hydroxyphenyl)methyl]piperazin-1-yl]methyl]phenol | Yes | Yes | Yes | No | No | Yes | No | No | Yes | No | No | Yes | Yes | Yes | Yes | No | No | Yes |
| Podocarpan-14beta-ol | No | No | Yes | No | Yes | No | Yes | Yes | Yes | Yes | No | No | No | No | Yes | No | No | Yes |
| Palmitic Acid | No | No | Yes | Yes | No | No | No | No | Yes | Yes | No | No | No | No | Yes | No | No | Yes |
| Succinic acid, hex-4-yn-3-yl pentyl ester | No | No | Yes | No | No | Yes | No | No | Yes | No | No | Yes | No | No | Yes | No | No | Yes |
| Methyl palmitate | No | No | Yes | Yes | Yes | Yes | No | No | Yes | No | No | Yes | No | No | Yes | No | No | Yes |
| 9-Octadecenoic acid | No | No | Yes | Yes | Yes | Yes | No | No | Yes | Yes | No | No | No | No | Yes | No | No | Yes |
| Oleic acid | No | No | Yes | Yes | Yes | Yes | No | No | Yes | Yes | No | No | No | No | Yes | No | No | Yes |
| Elaidic acid | No | No | Yes | Yes | Yes | Yes | No | No | Yes | Yes | No | No | No | No | Yes | No | No | Yes |
| Methyl elaidate | No | No | Yes | Yes | Yes | Yes | No | No | Yes | No | No | Yes | No | No | Yes | No | No | Yes |
| Methyl vaccenate | No | No | Yes | Yes | Yes | Yes | No | No | Yes | No | No | Yes | No | No | Yes | No | No | Yes |
| Methyl oleate | No | No | Yes | Yes | Yes | Yes | No | No | Yes | No | No | Yes | No | No | Yes | No | No | Yes |
| Methyl stearate | No | No | Yes | Yes | Yes | Yes | No | No | Yes | No | No | Yes | No | No | Yes | No | No | Yes |
| Stigmastan-3,5-diene | No | No | Yes | No | No | Yes | No | No | Yes | Yes | No | No | No | No | Yes | No | No | Yes |
| **Proportion of Agreement** |  |  | **92%** |  |  | **79%** |  |  | **52%** |  |  | **44%** |  |  | **93%** |  |  | **95%** |

**Additional File: Table 6c_Comparison of Excretion properties of the identified compounds using pkCSM and SwissADME tools**

| Compound | Excretion | | |
| --- | --- | --- | --- |
|  | pkCSM | SwissADME | Consensus |
|  | Renal OCT substrate | | |
| 1,3-Dichloropropane | No | N/A | N/A |
| Isophthalaldehyde | No | N/A | N/A |
| Terephthalaldehyde | No | N/A | N/A |
| 5-Indanol | No | N/A | N/A |
| 3,4-Dimethyl-3-cyclohexenylmethanal | No | N/A | N/A |
| Tridecane | No | N/A | N/A |
| Precocene I | No | N/A | N/A |
| 1,4-Di-tert-butylbenzene | No | N/A | N/A |
| 1,3-Di-tert-butylbenzene | No | N/A | N/A |
| Tetradecane | No | N/A | N/A |
| Humulene | No | N/A | N/A |
| beta-Patchoulene | No | N/A | N/A |
| 4a,5-Dimethyl-3-(prop-1-en-2-yl)-1,2,3,4,4a,5,6,7-octahydronaphthalene | No | N/A | N/A |
| Cedrene | No | N/A | N/A |
| (-)-alpha-Gurjunene | No | N/A | N/A |
| (+)-Aromadendrene | No | N/A | N/A |
| (+)-alpha-Longipinene | No | N/A | N/A |
| (+)-gamma-Cadinene | No | N/A | N/A |
| alpha-Cadinene | No | N/A | N/A |
| Aromadendrene | No | N/A | N/A |
| beta-Humulene | No | N/A | N/A |
| Naphthalene, 1,2,3,4,4a,5,6,8a-octahydro-7-methyl-4-methylene-1-(1-methylethyl)-, (1alpha,4aalpha,8aalpha)- | No | N/A | N/A |
| 3,7(11)-Eudesmadiene | No | N/A | N/A |
| 1,2,4a,5,6,8a-Hexahydro-1-isopropyl-4,7-dimethylnaphthalene | No | N/A | N/A |
| Cadina-1(10),4-diene | No | N/A | N/A |
| (-)-cis-beta-Elemene | No | N/A | N/A |
| alpha-Bergamotene | No | N/A | N/A |
| 2-Isopropenyl-1-methyl-4-(1-methylethylidene)-1-vinylcyclohexane | No | N/A | N/A |
| (+)-Cyclosativene | No | N/A | N/A |
| Epizonarene | No | N/A | N/A |
| (+)-alpha-Muurolene | No | N/A | N/A |
| 2-Isopropenyl-4a,8-dimethyl-1,2,3,4,4a,5,6,7-octahydronaphthalene | No | N/A | N/A |
| Eudesma-4(14),7(11)-diene | No | N/A | N/A |
| (+)-Helminthogermacrene | No | N/A | N/A |
| Naphthalene, 1,2,3,4,4a,5,6,8a-octahydro-4a,8-dimethyl-2-(1-methylethenyl)-, [2R-(2alpha,4aalpha,8abeta)]- | No | N/A | N/A |
| 8-Isopropenyl-1,5-dimethyl-cyclodeca-1,5-diene | No | N/A | N/A |
| 3,5-Di-tert-butylphenol | No | N/A | N/A |
| Patchoulane | No | N/A | N/A |
| 7R,8R-8-Hydroxy-4-isopropylidene-7-methylbicyclo [5.3.1]undec-1-ene | No | N/A | N/A |
| 1H-Benzocyclohepten-7-ol, 2,3,4,4a,5,6,7,8-octahydro-1,1,4a,7-tetramethyl-, cis- | No | N/A | N/A |
| 2-Amino-3,5-dibromopyridine | No | N/A | N/A |
| Octadecane | No | N/A | N/A |
| Nonadecane | No | N/A | N/A |
| 1,5,9-Cyclotetradecatriene, 1,5,9-trimethyl-12-(1-methylethenyl)- | Yes | N/A | N/A |
| Eicosane | No | N/A | N/A |
| Bis(2-ethylhexyl) phthalate | No | N/A | N/A |
| Diisooctyl phthalate | No | N/A | N/A |
| Di-n-2-propylpentylphthalate | No | N/A | N/A |
| 2,5-Ditert-butyl-4-[[4-[(2,5-ditert-butyl-4-hydroxyphenyl) methyl]piperazin-1-yl]methyl]phenol | No | N/A | N/A |
| Podocarpan-14beta-ol | No | N/A | N/A |
| Palmitic Acid | No | N/A | N/A |
| Succinic acid, hex-4-yn-3-yl pentyl ester | No | N/A | N/A |
| Methyl palmitate | No | N/A | N/A |
| 9-Octadecenoic acid | No | N/A | N/A |
| Oleic acid | No | N/A | N/A |
| Elaidic acid | No | N/A | N/A |
| Methyl elaidate | No | N/A | N/A |
| Methyl vaccenate | No | N/A | N/A |
| Methyl oleate | No | N/A | N/A |
| Methyl stearate | No | N/A | N/A |
| Stigmastan-3,5-diene | No | N/A | N/A |

**Additional File: Table 6c_Comparison of Toxicity properties of the identified compounds using pkCSM and SwissADME tools**

| Compound | Toxicity | | | | |
| --- | --- | --- | --- | --- | --- |
|  | pkCSM | | | SwissADME | Consensus |
|  | AMES toxicity | Hepatotoxicity | Skin Sensitisation | AMES/Hepatotoxicity/Skin Sensitisation | AMES/Hepatotoxicity/Skin Sensitisation |
| 1,3-Dichloropropane | Yes | No | No | N/A | N/A |
| Isophthalaldehyde | No | No | Yes | N/A | N/A |
| Terephthalaldehyde | No | No | Yes | N/A | N/A |
| 5-Indanol | No | Yes | Yes | N/A | N/A |
| 3,4-Dimethyl-3-cyclohexenylmethanal | No | No | Yes | N/A | N/A |
| Tridecane | No | No | Yes | N/A | N/A |
| Precocene I | No | No | Yes | N/A | N/A |
| 1,4-Di-tert-butylbenzene | No | No | Yes | N/A | N/A |
| 1,3-Di-tert-butylbenzene | No | No | Yes | N/A | N/A |
| Tetradecane | No | No | Yes | N/A | N/A |
| Humulene | No | No | Yes | N/A | N/A |
| beta-Patchoulene | No | No | No | N/A | N/A |
| 4a,5-Dimethyl-3-(prop-1-en-2-yl)-1,2,3,4,4a,5,6,7-octahydronaphthalene | No | No | Yes | N/A | N/A |
| Cedrene | No | No | No | N/A | N/A |
| (-)-alpha-Gurjunene | No | No | No | N/A | N/A |
| (+)-Aromadendrene | No | No | No | N/A | N/A |
| (+)-alpha-Longipinene | No | No | No | N/A | N/A |
| (+)-gamma-Cadinene | No | No | Yes | N/A | N/A |
| alpha-Cadinene | No | No | Yes | N/A | N/A |
| Aromadendrene | No | No | No | N/A | N/A |
| beta-Humulene | No | No | Yes | N/A | N/A |
| Naphthalene, 1,2,3,4,4a,5,6,8a-octahydro-7-methyl-4-methylene-1-(1-methylethyl)-, (1alpha,4aalpha,8aalpha)- | No | No | Yes | N/A | N/A |
| 3,7(11)-Eudesmadiene | No | No | Yes | N/A | N/A |
| 1,2,4a,5,6,8a-Hexahydro-1-isopropyl-4,7-dimethylnaphthalene | No | No | Yes | N/A | N/A |
| Cadina-1(10),4-diene | No | No | Yes | N/A | N/A |
| (-)-cis-beta-Elemene | No | No | Yes | N/A | N/A |
| alpha-Bergamotene | No | No | Yes | N/A | N/A |
| 2-Isopropenyl-1-methyl-4-(1-methylethylidene)-1-vinylcyclohexane | No | No | Yes | N/A | N/A |
| (+)-Cyclosativene | No | No | No | N/A | N/A |
| Epizonarene | No | No | Yes | N/A | N/A |
| (+)-alpha-Muurolene | No | No | Yes | N/A | N/A |
| 2-Isopropenyl-4a,8-dimethyl-1,2,3,4,4a,5,6,7-octahydronaphthalene | No | No | Yes | N/A | N/A |
| Eudesma-4(14),7(11)-diene | No | No | Yes | N/A | N/A |
| (+)-Helminthogermacrene | No | No | Yes | N/A | N/A |
| Naphthalene, 1,2,3,4,4a,5,6,8a-octahydro-4a,8-dimethyl-2-(1-methylethenyl)-, [2R-(2alpha,4aalpha,8abeta)]- | No | No | Yes | N/A | N/A |
| 8-Isopropenyl-1,5-dimethyl-cyclodeca-1,5-diene | No | No | Yes | N/A | N/A |
| 3,5-Di-tert-butylphenol | No | No | Yes | N/A | N/A |
| Patchoulane | No | No | No | N/A | N/A |
| 7R,8R-8-Hydroxy-4-isopropylidene-7-methylbicyclo[5.3.1]undec-1-ene | No | No | Yes | N/A | N/A |
| 1H-Benzocyclohepten-7-ol, 2,3,4,4a,5,6,7,8-octahydro-1,1,4a,7-tetramethyl-, cis- | No | No | Yes | N/A | N/A |
| 2-Amino-3,5-dibromopyridine | No | No | Yes | N/A | N/A |
| Octadecane | No | No | Yes | N/A | N/A |
| Nonadecane | No | No | Yes | N/A | N/A |
| 1,5,9-Cyclotetradecatriene, 1,5,9-trimethyl-12-(1-methylethenyl)- | No | No | Yes | N/A | N/A |
| Eicosane | No | No | Yes | N/A | N/A |
| Bis(2-ethylhexyl) phthalate | No | No | No | N/A | N/A |
| Diisooctyl phthalate | No | No | No | N/A | N/A |
| Di-n-2-propylpentylphthalate | No | No | No | N/A | N/A |
| 2,5-Ditert-butyl-4-[[4-[(2,5-ditert-butyl-4-hydroxyphenyl) methyl]piperazin-1-yl]methyl]phenol | Yes | Yes | No | N/A | N/A |
| Podocarpan-14beta-ol | No | No | Yes | N/A | N/A |
| Palmitic Acid | No | No | Yes | N/A | N/A |
| Succinic acid, hex-4-yn-3-yl pentyl ester | No | No | No | N/A | N/A |
| Methyl palmitate | No | No | Yes | N/A | N/A |
| 9-Octadecenoic acid | No | No | Yes | N/A | N/A |
| Oleic acid | No | No | Yes | N/A | N/A |
| Elaidic acid | No | No | Yes | N/A | N/A |
| Methyl elaidate | No | No | Yes | N/A | N/A |
| Methyl vaccenate | No | No | Yes | N/A | N/A |
| Methyl oleate | No | No | Yes | N/A | N/A |
| Methyl stearate | No | No | Yes | N/A | N/A |
| Stigmastan-3,5-diene | No | No | No | N/A | N/A |
