# Supplementary material for: GC–MS analysis, molecular docking, and pharmacokinetic studies of Multidentia crassa extracts’ compounds for analgesic and anti-inflammatory activities in dentistry
Source: Sci Rep. 2024 Jan 22;14:1876. doi: 10.1038/s41598-023-47737-x (PMC10803350; doi:10.1038/s41598-023-47737-x)
Supplement: Supplementary file 5 — Supplementary Table 7. [file 41598_2023_47737_MOESM5_ESM.docx]

**Additional File: Table 5_Molecular Docking results for the phytocompounds with their binding energies for each target protein**

| *S/N* |  | *Compound name* | *Binding energy of ligands against analgesic targets(kcal/mol)* | | | *Binding energy of ligands against anti-inflammatory targets(kcal/mol)* |
| --- | --- | --- | --- | --- | --- | --- |
|  | *Solvent* |  | *Cold Sensor Protein* | *Cyclooxyegnase-2* | *P2X3 Purinergic receptor* | *Interleukin-1* |
| 1 | Reference standard | Ibuprofen | -7 | -8 | -5.8 | - |
| 1 | DICHLOROMETHANE | 2-Pyridinamine, 3,5-dibromo- | -5.7 | -5.4 | -4.2 | -5.3 |
| 2 |  | 1H-Benzocyclohepten-7-ol, 2,3,4,4a,5,6,7,8-octahydro-1,1,4a,7-tetramethyl-, cis- | -6.7 | -7.2 | -6.1 | -6.4 |
| **3** |  | ***7R,8R-8-Hydroxy-4-isopropylidene-7-methylbicyclo[5.3.1]undec-1-ene** | **-7.2** | **-7.1** | **-6.3** | **-7** |
| 4 |  | Phenol, 3,5-bis(1,1-dimethylethyl) | -7 | -7.1 | -5.5 | -6.8 |
| 5 |  | Precocene I | -6.8 | -7.2 | -5.5 | -6.9 |
| 6 |  | 3-Cyclohexen-1-carboxaldehyde, 3,4-dimethyl- | -6.9 | -7.3 | -4.6 | -5.6 |
| **7** |  | ***1H-Inden-5-ol, 2,3-dihydro-** | **-7.5** | **-6.8** | **-5.3** | **-6.4** |
| 8 |  | 1,4-Benzenedicarboxaldehyde | -6.2 | -5.8 | -4.8 | -5.3 |
| 9 |  | Isophthalaldehyde | -6.7 | -5.8 | -4.9 | -5.1 |
| 10 |  | Propane, 1,3-dichloro- | -3.6 | -3.8 | -2.9 | -3.1 |
| 11 | METHANOL | Octadec-9-enoic acid | -6.6 | - | - | - |
| 12 |  | n-Hexadecanoic acid | -6.5 | - | - | - |
| 13 |  | Hexadecanoic acid, methyl ester | -6.7 | - | - | - |
| 14 |  | Methyl stearate | -6.1 | -5.5 | - | -5.9 |
| 15 |  | Oleic Acid | -6.6 | -6 | - | -6.4 |
| **16** |  | ***Stigmastan-3,5-diene** | **-10.1** | **-9.7** | **-** | **-7.9** |
| 17 |  | 9-Octadecenoic acid, (E)- | -6.9 | -6.5 | - | - |
| 18 |  | 9-Octadecenoic acid, methyl ester,(E)- | -6.4 | -5.8 | - | -5.7 |
| 19 |  | 11-Octadecenoic acid, methyl ester | -6.3 | -5.9 | - | -5.6 |
| 20 |  | 9-Octadecenoic acid (Z)-, methyl ester | -6.3 | -5.9 | - | - |
| **21** |  | ***1-Phenanthrenol, tetradecahydro-4b,8,8-trimethyl-, [1R-(1.alpha.,4a.beta.,4b.alpha.,8a.beta.,10a.alpha.)]-** | **-8.2** | **-8.1** | **-** | **-** |
| 22 |  | Succinic acid, hex-4-yn-3-yl pentyl Ester | -5.7 | -6.4 | -4.7 | -5.5 |

**-** Means it produced invalid docking output (so no results were produced, applying similarly to the rest of cells with dashes.

* compounds that showed the best binding energies
