# Supplementary material for: GC–MS analysis, molecular docking, and pharmacokinetic studies of Multidentia crassa extracts’ compounds for analgesic and anti-inflammatory activities in dentistry
Source: Sci Rep. 2024 Jan 22;14:1876. doi: 10.1038/s41598-023-47737-x (PMC10803350; doi:10.1038/s41598-023-47737-x)
Supplement: Supplementary file 6 — Supplementary Table 8. [file 41598_2023_47737_MOESM6_ESM.docx]

**Additional File Table 8: Drug target chemical interactions**

| **Ligand** | **Target protein** | **Binding residue** | **Bond length (A°)** | **Type of interactions** |
| --- | --- | --- | --- | --- |
| **Stigmastan-3,5-diene** | Cold Sensor Protein (7D4Q or 7WDB) | A:TRP577  A:PHE576  A:PHE580  A:TYR524  D:Val610  D:VAL614 | 3.49  4.58  3.69  3.93  3.61  3.67 | Pi-Alkyl  Pi-Alkyl  Van der Waals  Van der Waals  Alkyl  Alkyl |
|  | Cyclooxyegnase-2 | B:LEU145  B:PHE142  B:TRP139  A:PHE142  A:HIS226 | 4.36  3.83  4.34  4.53  4.69 | Pi-Alkyl  Pi-Alkyl  Van der Waals  Van der Waals  Alkyl |
|  | Interleukin-1 | R:ALA125  R:ILE130  R:VAL212 | 4.30  5.36  3.34 | Pi-Alkyl  Pi-Alkyl  Alkyl |
| 1-Phenanthrenol, tetradecahydro-4b,8,8-trimethyl-, [1R-(1.alpha.,4a.beta.,4b.alpha.,8a.beta.,10a.alpha.)]- | Cold Sensor Protein (7D4Q or 7WDB) | A:PHE576  A:LEU528  A:LEU572  D:ALA602  D:THR603 | 3.59  4.58  3.69  3.93  3.61 | Pi-Sigma  Pi-Alkyl  Van der Waals  Alkyl  Alkyl |
|  | Cyclooxyegnase-2 | A:GLY536  B:PHE142  A:LEU145  B:LEU145 | 4.23  3.19  4.14  4.47 | Hydrogen Bond  Pi-Alkyl  Van der Waals  Alkyl |
| 7R,8R-8-Hydroxy-4-isopropylidene-7-methylbicyclo[5.3.1]undec-1-ene | Cold Sensor Protein (7D4Q or 7WDB ) | A:VAL610  B:VAL579  A:VAL614  B:PHE580  B:PHE576  B:LEU521 | 3.11  4.87  3.69  3.53  3.61  3.49 | Pi-Alkyl  Pi-Alkyl  Van der Waals  Van der Waals  Alkyl  Alkyl |
|  | Cyclooxyegnase-2 | A:TYR385  A:SER530  A:LEU352  A:ALA527 | 4.11  3.33  4.85  4.47 | Hydrogen Bond  Hydrogen Bond  Van der Waals  Alkyl |
|  | P2X3 Purinergic Receptor | A:ARG295  A:TRP152  A:LEU297  A:ILE | 3.87  3.76  4.95  4.14 | Hydrogen Bond  Pi-Alkyl  Van der Waals  Alkyl |
|  | Interleukin-1 | R:ALA125  R:ILE130  R:TRP140 | 4.44  5.21  3.91 | Pi-Alkyl  Pi-Alkyl  Alkyl |
| 1H-Inden-5-ol, 2,3-dihydro- | Cold Sensor Protein (7D4Q or 7WDB) | A:GLU138  A:TYR102  A:LYS106  A:ARG71  A:ARG105  A:GLU79 | 2.99  4.11  3.72  3.93  3.82  3.43 | Hydrogen Bond  Pi-Alkyl  Van der Waals  Van der Waals  Pi-Anion  Pi-Cation |
|  | Cyclooxyegnase-2 | A:THR206  A:ALA202  A:LEU391  A:HIS388  A:ALA199 | 3.72  3.22  3.65  3.76  3.81 | Hydrogen Bond  Alkyl  Pi-Alkyl  Pi-Alkyl  Alkyl |
|  | P2X3 Purinergic receptor | A:THR172  A:PHE174  A:ILE215  A:LYS65 | 4.21  3.53  3.16  3.48 | Hydrogen Bond  Pi-Alkyl  Pi-Alkyl  Alkyl |
|  | Interleukin-1 | R:ARG25  R:LEU15  R:PRO28  R:ILE92 | 2.98  3.22  3.15  3.44 | Hydrogen Bond  Pi-Alkyl  Alkyl  Alkyl |
